# Prevalence of musculoskeletal disorders among dental students: A systematic review and meta-analysis

**DOI:** 10.1016/j.heliyon.2023.e19956

**Published:** 2023-09-11

**Authors:** Manuel Barbosa Almeida, Rita Póvoa, Duarte Tavares, Paula Moleirinho Alves, Raúl Oliveira

**Affiliations:** a Neuromuscular Research Lab, Interdisciplinary Centre for the Study of Human Performance (CIPER), Faculty of Human Kinetics, University of Lisbon, Estrada da Costa, Cruz Quebrada, 1499-002, Dafundo, Oeiras, Portugal; b Department of Physiotherapy, Egas Moniz School of Health & Science, Campus Universitario, Quinta da Granja, 2829-511, Monte da Caparica, Almada, Portugal; c Integrative Movement and Networking Systems Laboratory (INMOV-NET LAB) - Egas Moniz Center for Interdisciplinary Research (CiiEM), Egas Moniz School of Health & Science, Campus Universitário, Quinta da Granja, 2829-511, Caparica, Almada, Portugal; d Physical and Functional Assessment Laboratory in Physiotherapy (LAFFFi) - Egas Moniz Center for Interdisciplinary Research (CiiEM), Egas Moniz School of Health & Science, Campus Universitário, Quinta da Granja, 2829-511, Caparica, Almada, Portugal; e Egas Moniz Center for Interdisciplinary Research (CiiEM), Egas Moniz School of Health & Science, Campus Universitário, Quinta da Granja, 2829-511, Caparica, Almada, Portugal; f Regional Health Administration of Lisbon and Tagus Valley, P.I., Av. Estados Unidos da América nº 77, 1749-096, Lisboa, Portugal

## Abstract

**Objectives:**

This study aimed to determine the prevalence of work-related musculoskeletal disorders (WMSDs) in dental students and analyze the potential associated risk factors.

**Methods:**

This review was registered in PROSPERO with the number CRD42022349864. We performed a meta-analysis calculating event rates with relative 95% confidence intervals for each body region. Two investigators systematically searched Cochrane, Pubmed, Scopus, and EBSCO databases following the Preferred Reporting Items for Systematic Reviews and Meta-Analyses (PRISMA).

**Results:**

Sixteen studies, with 3761 dental students, were included. The highest 7-day prevalence was in the lower back (27.2%; 95% CI 20–35), neck (27%; 95% CI 19.1–35.8), and upper back (24.2%; 95% CI 17.2–32). Yearly occurrence was mainly in the neck (51%; 95% CI 41–61), followed by shoulders (45.3%; 95% CI 37.6–53.1) and lower back (42%; 95% CI 34.1–50.2) and a fraction of these reported that symptoms in lower-back (15.2%; 95% CI 12.1–18.5), neck (13.9%; 95% CI 10.6–17.5) and shoulders (12.2%; 95% CI 8.7–16.3) affected work or normal activities. Associated contributing factors include female sex, poor posture habits, inadequate ergonomics knowledge, sedentary lifestyle, high physical activity levels, poor quality of life, and smoking. In contrast, engaging in physical exercise has positively impacted mitigating the risk of musculoskeletal disorders.

**Conclusions:**

WMSDs have a high prevalence among dental students, particularly in the cervicothoracic, lumbar, and shoulder regions, having a significant impact since training years. Further research with a multidimensional approach with psychosocial and physical assessments is recommended to understand this issue thoroughly.

## 1 Introduction

In recent years, work-related musculoskeletal disorders (WMSDs) have become one of the most critical health issues among healthcare workers [1], especially for health professions like dentists, with up to 95,8% developing WMSDs in their lifetime and 92% reporting musculoskeletal pain in the last year [[1], [2], [3]]. Other studies determined that this issue could begin early in dentists' careers, with a prevalence among students between 44% and 93% [2,[4], [5], [6], [7], [8], [9]], with an increasing number of preclinical dental students voicing concerns about WMSDs [3]. In two literature reviews of the general health of dentists, WMSDs were identified as a significant issue for the profession [1], as they contribute considerably to sick leave, reduced productivity, and terminating clinical practice earlier than they desired [1,3].

WMSDs comprise a diverse range of inflammatory and degenerative conditions. They are defined by the World Health Organization (WHO) as a disorder of muscles, ligaments, tendons, joints, nerves, and bones not directly resulting from an acute or instantaneous event (e.g., slips or falls). These disorders are defined as discomfort, disability impairment, or persistent pain in the locomotor system. They usually come under the umbrella term work-related musculoskeletal disorders when the work environment promotes its development or aggravation [1,[10], [11], [12]].

Current literature highlights a greater risk of dental professionals developing musculoskeletal disorders or symptoms than the general population [1,2]. A wide variety of causative factors have been associated with WMSDs. Although increasing evidence is suggesting that psychosocial factors may be associated with the prevalence of WMSDs, the physical burden of clinical work associated with incorrect postures or poor body mechanics have been described as the major factors associated with WMSDs referred by dental health workers [1,3,5,13,14]. The oral cavity represents a minimal work area, which is challenging to access and navigate while providing dental care, promoting static, asymmetrical, and unsuitable positions [2]. A dentist's work position implies leaning their head towards patients with arms distant from the body and continuous trunk rotation while maintaining high attention and concentration for long periods. Repeated use of these positions results in excessive and continued pressure on musculoskeletal structures in the neck, shoulders, trunk, and waist, exacerbating and highlighting the WMSDs impact in this profession and ultimately leading to reduced working efficiency and early disabled dentists [2,5,6,15,16]. Several dental procedures, such as filling a cavity or preparing a root canal require static body postures [17], defined as body positions maintained for more than 4 s [18]. A kinematic analysis of work-related musculoskeletal loading of the trunk determined static positions of the dentist's head and trunk generally remained for 27.4% and 23.6% of the treatment time, respectively [2]. A higher level of muscle activity, a strained posture, and repetitive movements are the most recognized risk factors for developing WMSDs [19,20].

Despite the growing research on the development of musculoskeletal disorders related to dentists' clinical practice, a systematic review was not yet conducted to determine WMSDs prevalence in dental students. These students undergo a distinct professional development phase, including intensive training, clinical practice, and skill acquisition. Methodic and valuable information for developing targeted interventions and preventive strategies that address the unique challenges faced during the educational phase is needed. Dental students represent the future workforce in dentistry. Their well-being and occupational health during training affect their future professional lives. While the focus is on dental students, the systematic review's findings may have broader implications, helping to understand WMSDs in dental professionals better and contributing to the overall body of knowledge in the field.

Therefore, this systematic review and meta-analysis aimed to determine the prevalence of work-related musculoskeletal disorders in dental students and analyze its associated risk factors.

## 2 Materials and methods

This systematic review protocol was prospectively registered on PROSPERO with the number: CRD42022349864 and did not require ethics approval. This review is reported following the Preferred Reporting Items for Systematic Reviews and Meta-Analyses (PRISMA) [21].

### 2.1 Inclusion criteria

We included cross-sectional, prospective, or longitudinal design studies conducted with dental students, with the prevalence of musculoskeletal disorders assessed using a standard and valid measure published after 2000 in English or Portuguese in peer-reviewed journals.

### 2.2 Exclusion criteria

Studies that included students and professional dentists in the same group with physically impaired participants, that excluded participants based on sociodemographic features, no peer-review journals, letters to authors, and opinion articles were excluded.

### 2.3 Information sources

We systematically searched PubMed, COCHRANE, EBSCO, and Scopus electronic databases since their inception until August 2022. The search strategy was made in Pubmed and adapted to other databases.

### 2.4 Search strategy

Terms used for the search strategy included at least one term from the following base concepts: student or undergraduate; dentist or dental; musculoskeletal or orthopedic or orthopaedic; symptom or disorder or dysfunction or injur*; prevalence. These terms should be in the title, abstract, or as an associated MeSH (Medical Subject Headings) term (Appendix 1). The search was repeated before the final analysis.

### 2.5 Study selection

We used Covidence® software (Veritas Health Innovation Ltd, Melbourne, Australia) for systematic review management. Two authors (MA and RP) screened all titles and abstracts identified through electronic databases searching and reference list scanning, removing the duplicates. The eligible articles progressed to full-text screening to determine their inclusion or exclusion. After the inclusion list was cross-checked between authors, a third author (RO) resolved any discrepancies.

### 2.6 Data collection and quality appraisal

The authors collaborated on developing and refining a data collection sheet. The sheet was pilot tested in studies with similar designs to the included articles. The extracted data covered information about the authors, publication year, study design, appraisal tool score, country, year of study enrollment, questionnaire used, population characteristics, and results for each body region. Two authors (MA and RP) independently registered the data from the studies, including their critical appraisal, and any inconsistencies were discussed among the review authors with the assistance of a third author (RO) to resolve any remaining discrepancies. Authors were contacted by email to provide additional data if needed.

Studies critical appraisal were made using the AXIS tool [22] for cross-sectional studies and the Critical Appraisal Skills Programme (CASP) instrument [23] for prospective designs.

Instrument scores were converted into percentages to rate studies as good (≥75%), fair (50–74%), or poor (<50%) based on both reviewers' assessment of the risk of bias.

The level of evidence was evaluated using the Grading Recommendations Assessment, Development, and Evaluation (GRADE) methodology. We applied the GRADE guidelines for evidence related to prognosis factors and assessed five domains: risk of bias, imprecision, inconsistency, indirectness, and publication bias. The quality of evidence was rated on a spectrum from high to very low, based on the confidence level that the estimated risk associated with the prognostic factor accurately reflects the true variation.

### 2.7 Statistical analysis

Reported prevalence was quantified as event rates in 7 days, 12 months, and 12 months affecting work or activity, allowing the determination of WMSDs occurrence for each period and body region. Proportion meta-analyses were performed using the MedCalc® Statistical Software version 20.215 (MedCalc Software Ltd, Ostend, Belgium). The meta-analysis included all studies with comparable outcomes. Event rates and respective 95% Confidence Intervals (CI) were calculated for each body region and period. We calculated the Cochran Q and the I^2^ to determine the heterogeneity and inconsistency of studies and used a random effects model if heterogeneity was confirmed (Cochran Q p < 0.10 and I^2^ > 50%). We used Egger's and Begg's tests to assess the presence of publication bias for each outcome. P-values <0.05 were considered statistically significant.

## 3 Results

**Fig. 1:**
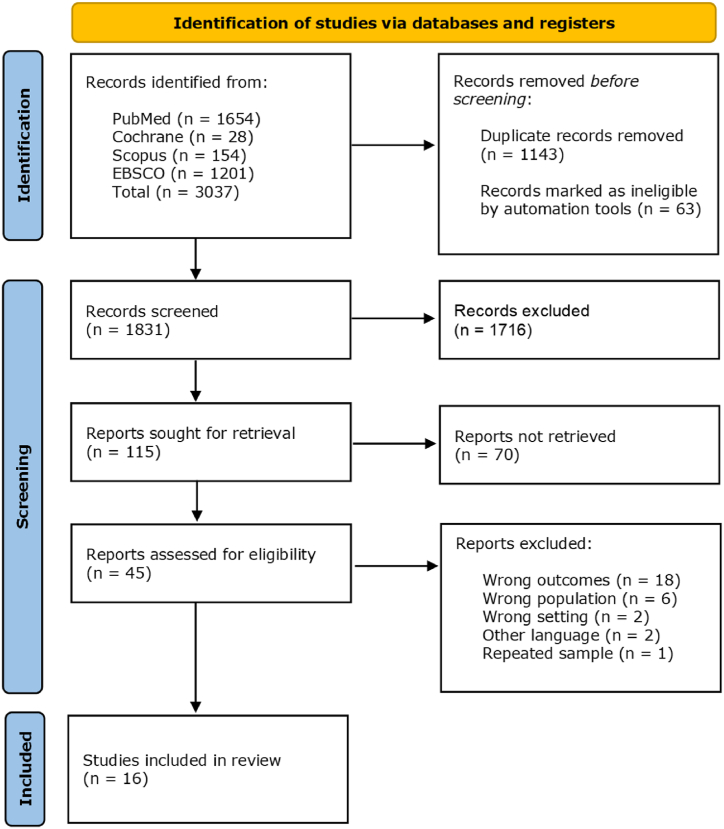
Systematic review flow chart.

We identified 3037 studies in our research with 1206 duplicates or marked as ineligible by automation tools removed before screening. The remaining 1831 records were screened for title, and 1716 were excluded. A total of 115 abstracts were reviewed, and 45 articles progressed to full-text analysis, where 29 more studies were excluded due to having non-comparable outcomes (18), studying the wrong population (6), having the wrong methods (2), written in other languages (2) and with a repeated sample of other included study (1). The resulting 16 studies integrated the systematic review and meta-analysis [6,7,[24], [25], [26], [27], [28], [29], [30], [31], [32], [33], [34], [35], [36], [37]]. The review flowchart [21] is represented in Fig. 1. These studies were published between 2009 and 2022. The authors used a cross-sectional design in 15 publications, and the remaining one by Kápitan et al. [27] had a prospective design, but only the data from the year with clinical training were retrieved for our review.

**Fig. 2:**
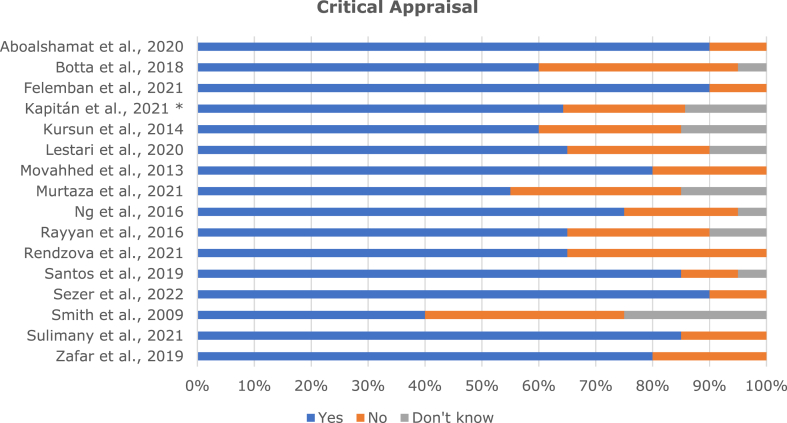
Critical appraisal details of included studies. Note. *We used CASP to critically appraise Kapitán et al., 2021 since it was a prospective study.

The total sample of dental students was 3615, with 2205 females (61%) and 1410 males (39%) with a mean age of 23.7 ± 1,6 years. Regarding Body Mass Index (BMI), 10% were underweight (<18.5 kg/m^2^), 63,4% were normal (18.5–24.9 kg/m^2^), and 26,6% were overweight (>25 kg/m^2^). Thirteen studies [6,7,[24], [25], [26], [27],^29^,[31], [32], [33], [34], [35], [36]] used the Nordic Musculoskeletal Questionnaire (NMQ) to assess the prevalence of WMSDs, one [37] used the self-reported occurrence of musculoskeletal complaints (OMC), one [30] used the Questionnaire of Body Pain (QBP) and one [28] used a Musculoskeletal Disorders Resulting from Clinical Practice (MDRCP) inquiry. The detailed data of included studies is described in Table 1. Critical appraisal scores rated as good (≥75%), fair (50–74%), or poor (<50%) are detailed in Fig. 2.

**Table 1:** Studies characteristics.

Authors	Year	Country	Design	Sample size	**Age** (Years±SD)	Sex		Instrument	Critical appraisal
						M	F		
Aboalshamat et al. [6]	2020	Saudi Arabia	C–S	274	23.5 ± 2.8	106	168	NMQ	Good
Botta et al. [24]	2018	USA	C–S	145	–	67	78	NMQ	Fair
Felemban et al. [26]	2021	Saudi Arabia	C–S	377	22.8 ± 1.4	145	232	NMQ	Good
Kapitán et al. [27]	2021	Czechia	P	73	23.6 ± 1.1	20	53	NMQ	Good
Kurşun et al. [28]	2014	Turkey	C–S	264	24	158	106	MDRCP	Fair
Lestari et al. [29]	2020	Indonesia	C–S	102	–	20	82	NMQ	Fair
Movahhed et al. [30]	2013	Iran	C–S	177	23.6 ± 3	87	90	QBP	Good
Murtaza et al. [31]	2021	Pakistan	C–S	422	–	145	277	NMQ	Fair
Ng et al. [7]	2016	Australia	C–S	136	–	58	78	NMQ	Good
Rayyan et al. [32]	2016	Saudi Arabia	C–S	191	–	0	191	NMQ	Fair
Rendzova et al. [33]	2021	Macedonia	C–S	116	22.6 ± 1.5	33	83	NMQ	Fair
Santos et al. [25]	2019	Brasil	C–S	241	22.3 ± 2	90	151	NMQ	Good
Sezer et al. [34]	2022	Turkey	C–S	105	23.2 ± 1.7	36	69	NMQ	Good
Smith et al. [35]	2009	Australia	C–S	56	22.5 ± 4.7	32	24	NMQ	Poor
Sulimany et al. [36]	2021	Saudi Arabia	C–S	794	24.8 ± 1.2	359	435	NMQ	Good
Zafar et al. [37]	2019	Saudi Arabia	C–S	142	–	54	88	OMC	Good

Note. C–S=Cross-sectional; F=Female; M = Male; MDRCP = Musculoskeletal Disorders Resulting from Clinical Practice; NMQ=Nordic Musculoskeletal; OMC=Occurrence of Musculoskeletal Complaints; P=Prospective; QBP = Questionnaire of Body Pain; SD=Standard deviation.

A meta-analysis was performed with the included studies for the prevalence of WMSDs in the last seven days, last 12 months, and last 12 months affecting work or normal activities on nine identified body regions. For self-reported symptoms in the last seven days, the lower back had the highest prevalence, with 27.2%, followed by the neck (27%), upper back (24.2%), and shoulders (19.6%). Other regions had lower rates starting with hands/wrists (12%), knees (11.5%), ankles/feet (10.3%), hips/thighs (8.9%), and elbows (3.9%). In the last 12 months, we observed higher occurrence rates starting by the neck with 51%, followed by shoulders (45.3%), lower back (42%), hands/wrists (40.1%), and upper back (38.9%). Ankles/feet with 19.6%, knees (18.1%), hips/thighs (13.3%), and elbows (7.8%) had lower prevalence results.

At least one in every seven students (15.2%) that reported lower back symptoms in the last year were forced to pause clinical practice or everyday activities. Neck (13.9%), shoulders (12.2%), upper back (11%), hands/wrists (8.8%), knees (8%), ankles/feet (5.9%), hips/thighs (4.2%) and elbows (3.3%) were other body regions with symptoms that affected daily activities.

The forest plots in Table 2, Table 3, Table 4 display the results of meta-analyses conducted on nine different musculoskeletal body regions, grouped into the three body regions with higher occurrences in the last seven days, last 12 months, and last 12 months affecting work or normal, respectively. The remaining data is available in the supplementary material (Appendix 2). All meta-analyses showed significant heterogeneity, indicated by an I^2^ value greater than 50%, except for knee prevalence affecting work or activity in the previous year.

**Table 2:**
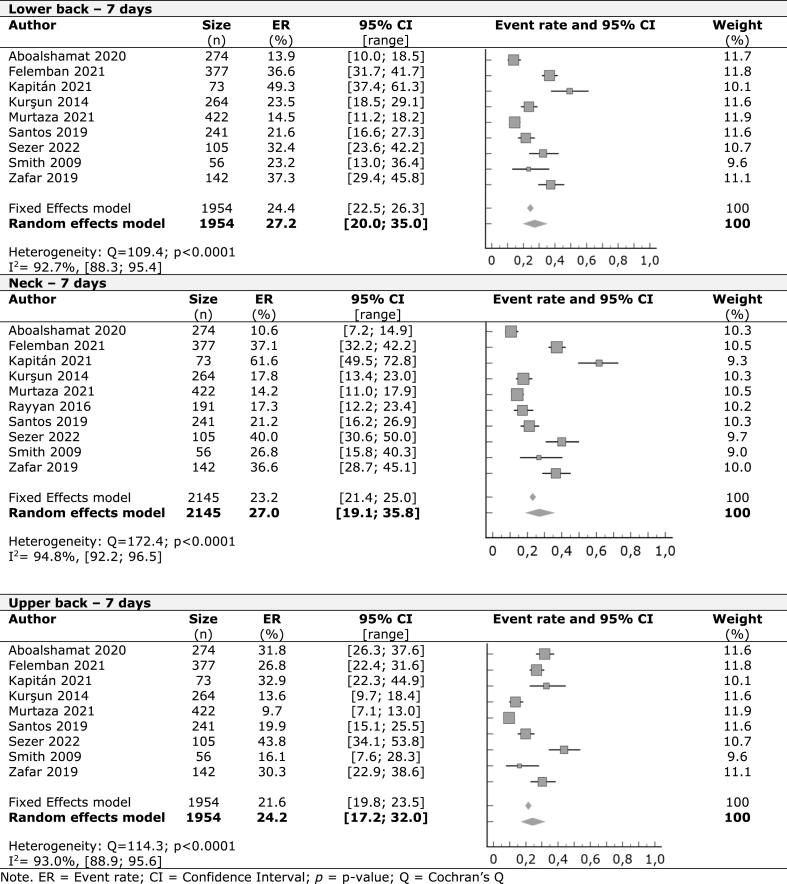
Work-related musculoskeletal disorders in the previous seven days – Regions with major prevalence.

**Table 3:**
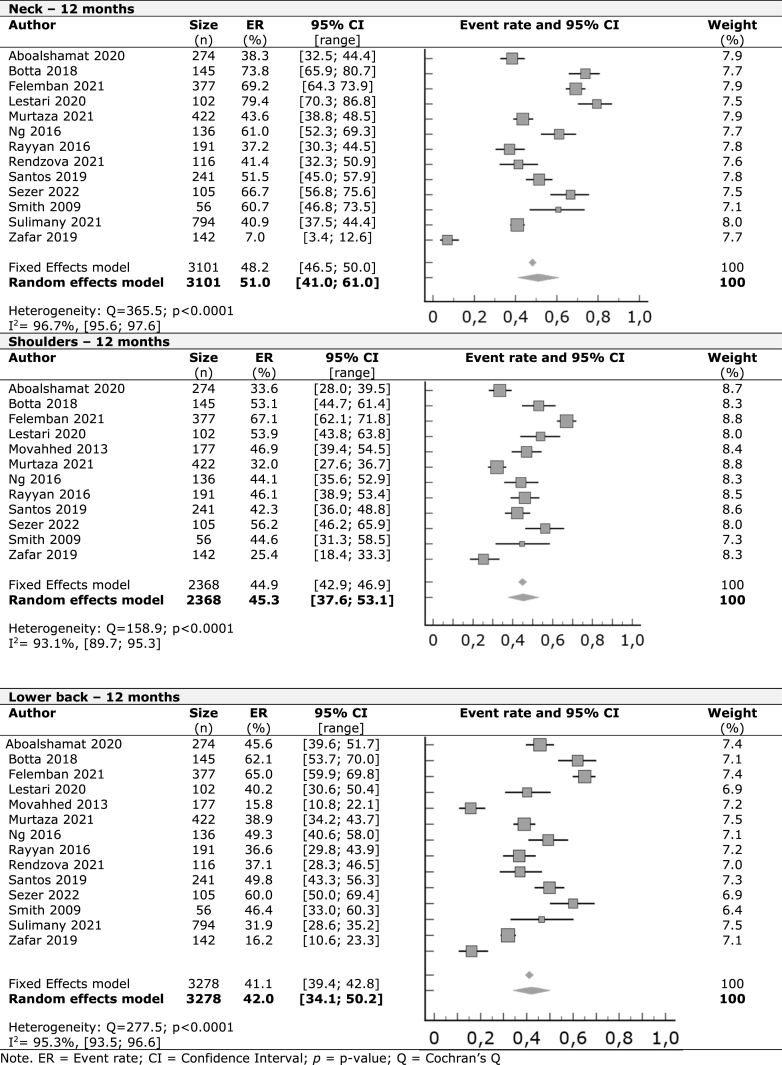
Work-related musculoskeletal disorders in the previous 12 months – Regions with major prevalence.

**Table 4:**
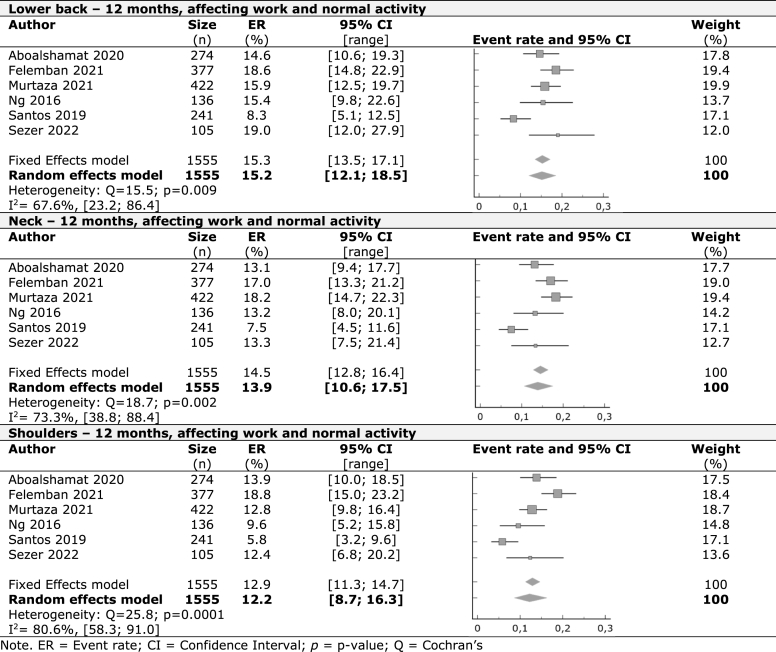
Work-related musculoskeletal disorders in the previous 12 months affecting work or normal activities – Regions with major prevalence.

The systematic review showed that all the included studies did not assess the duration and intensity of symptoms, leaving a gap in understanding the prevalence of symptoms in various body regions and at different time intervals. However, some studies reported a significant relationship between work-related musculoskeletal disorders and sociodemographic characteristics of dental students, particularly for female students in five studies [26,28,29,36,37] and for males in one study [6]. The results also indicated that several studies explored the postures and movements of dental students [7,29,31,33,35,36] as identified factors that contribute to WMSDs, such as lack of ergonomics knowledge [24,37], sedentary lifestyle [29,31], high physical activity [27], poor quality of life [34], and smoking [29]. On the other hand, the results showed that physical exercise had a protective effect on reducing the risk of WMSDs [26,30].

### 3.1 Meta-regression

A meta-regression could not be conducted because of the scarce number of studies that focused on the characteristics of dental students and the heterogeneity of its reporting.

### 3.2 Level of evidence

The results of our meta-analysis indicate an overall quality of evidence between low and very low for all the body regions across the three time periods assessed. There is a fair degree of confidence that clinical practice among dental students is associated with variations in risk. Only observational studies were included in the review, and their baseline level of evidence in the GRADE scaling was low due to their design. The high heterogeneity (I^2^>50%) observed in almost all body regions and the wide 95% Confidence Intervals (95% CI range difference >50% event rate) resulted in a downgrade of their level of evidence. However, most of the included studies had a good critical appraisal for each body region. Egger's and Begg's tests in all meta-analyses confirmed the inexistence of evidence of publication bias among the included studies, preventing additional downgrades. Detailed data on certainty assessment and GRADE level of evidence is in the supplementary material (Appendix 3).

## 4 Discussion

Work-related musculoskeletal disorders have recently emerged as a significant health concern among healthcare workers, particularly for health professionals such as dentists [1]. With WMSDs reported rates as high as 95.8% during their working careers [[1], [2], [3]], concerns arise about their affected areas, causes, risk factors, economic burden, and prevention.

Although there has been a growing body of research on the development of musculoskeletal disorders related to dentists' clinical practice, this systematic review and meta-analysis intend to fill a knowledge gap by determining the prevalence of work-related musculoskeletal disorders in dental students and analyze the potential associated risk factors, as previously recommended in a systematic review with dental professionals [1].

In this study, we collected data on the occurrence rates of work-related musculoskeletal disorders over a 7-day and 12-month follow-up period and the prevalence of disorders that prevented regular activity or work in the previous year.

According to our results on the prevalence of WMSDs in the past seven days, the lower back, neck, and upper back were the main areas of complaint. Given the nature of the students' clinical practice, these results were anticipated. The repetitive and prolonged efforts during dental procedures can lead to sustained static postures and repetitive movements, which are recognized risk factors for developing WMSDs [1,3,5,13,14]. No equivalent data is available from existing reviews on the reported symptoms of professional dentists over one week. Still, our results indicate similar prevalence rates in comparable studies with dentists, with the lower back ranging from 25.4% to 30%, the neck from 22% to 38.3%, the upper back at 15%, and the shoulders from 6.7% to 18.6% [38,39].

In a retrospective one-year spanning, we continue to observe a higher rate of reported disorders in the trunk and upper limbs. The neck had the highest prevalence rate, followed by the shoulder, lower back, hand/wrists, and upper back. Compared to a meta-analysis of professional dentists [16], we found slightly different results for prevalence rates of musculoskeletal disorders. While all body regions had higher rates among students, the neck and elbows showed similar rates between both groups. Students had 51% in the neck, 45.3% in the shoulder, and 42% in the lower back, which is consistent with the mechanical overload characteristic of the dental profession working posture, even from their training years.

Our findings challenge the conventional notion that extended practice and repeated movements result in a higher incidence of musculoskeletal disorders among professionals, providing novel insights into the understanding of this issue. The higher prevalence of musculoskeletal disorders among students compared to professional dentists could be due to several factors. One possible explanation is that students may not fully develop the proper technique and ergonomic strategies for dental procedures, leading to greater physical strain on their bodies. This could result in higher rates of musculoskeletal disorders in areas such as the shoulder, back, and hands, commonly used in dental procedures. Additionally, students may take longer to perform the same procedures as professional dentists, further exacerbating the physical demand on their bodies. The high occurrence rates of musculoskeletal disorders in dental students are concerning, particularly compared to the prevalence rates reported for the general population in the same age group. For instance, while the prevalence of WMSDs in the general population aged 20–30 years typically ranges from 9% for knee pain to 19% for low back pain [[40], [41], [42]], dental students have been found to experience even higher prevalence rates, with the most affected body regions surpassing the corresponding rates in the general population with some being affected two or three times more than the reference values.

It's also possible that students may not have received proper training or education on ergonomics and posture during dental procedures, which could contribute to the development of musculoskeletal disorders. Furthermore, students may be more likely to engage in behaviors that increase the risk of musculoskeletal disorders, such as sitting for long periods or engaging in repetitive movements without proper breaks or precautions. This highlights the importance of further investigating preventive ergonomic strategies while working.

Like the weekly data, the prevalence of conditions affecting work and daily activities over the past 12 months has been poorly documented in comparable studies and reviews among dentists. Still, our results indicate low levels of impairment observed among students, with 15.2% of students who reported lower back symptoms in the last year being forced to interrupt their clinical practice or everyday activities and with similar occurrence in the neck (13.9%) and shoulders (12.2%), suggesting that despite their reported musculoskeletal disorders, they are able to continue with their clinical practice and daily activities with minimal disruption. This could potentially be due to their relatively young age and ability to recover faster, and it is also possible that these conditions and activity implications may worsen as students progress in their careers. However, further investigation into the potential role of motor behavior variables, such as strength, muscle activity, postural control, and psychological variables like stress and anxiety, could be important in determining the extent of musculoskeletal symptoms and associated factors. Including these variables in future studies may provide a more comprehensive understanding of the long-term impact of musculoskeletal disorders on dentists' health and work performance.

We were unable to perform a meta-regression, but some of the included studies still provide valuable insights into the factors that could contribute to WMSDs in dental students. Several authors [26,28,29,36,37] noted a relationship between WMSDs and female sex. One of the indicated reasons for this could be that women are more health-conscious than males, therefore, more likely to report their symptoms. Another explanation lies in the sexual dimorphism, the differences in muscle tone, and the greater energy needs for females to generate the necessary force for their clinical tasks making them less resistant to chronic musculoskeletal tension. This highlights the need for targeted interventions to reduce the risk of WMSDs in this population. Additionally, the finding that physical exercise has a positive impact on reducing the risk of WMSDs is particularly noteworthy. This result reinforces the importance of promoting physical activity and exercise among dental students to reduce the risk of developing WMSDs. Future studies should explore the most effective types and duration of physical exercise for reducing the risk of WMSDs in this population and the potential mechanisms by which physical exercise reduces the risk of WMSDs. Ultimately, this information could inform the development of effective prevention strategies to reduce the burden of WMSDs on dental students.

Our findings highlight the significant burden of WMSDs on dental students and the need for effective interventions to prevent and manage these conditions. The repetitive and prolonged nature of dental procedures can result in prolonged static postures and repetitive movements, which are known risk factors for developing WMSDs. These findings suggest that dental students are at a high risk of developing WMSDs and that preventive measures should be implemented to reduce this risk.

The results of this study have important implications for dental education and training. Dental schools should provide students with appropriate training and resources to minimize the risk of WMSDs, such as ergonomic education and access to ergonomic equipment, and consider promoting and integrating a specific exercise approach within these students' training to reduce the reported occurrence rates. In addition, regular monitoring and assessment of WMSDs should be incorporated into the curriculum to ensure that students are aware of the risk factors and can take steps to prevent and manage these conditions. Given the multifaceted nature of WMSDs and the complexity of the dental profession, a combined implementation of the proposed preventive strategies may be more effective. Therefore, future efforts should focus on evaluating the actual effectiveness of these preventive measures for further standardizing their implementation.

All the included studies used self-reported questionnaires. Despite being an affordable and accessible way to gauge the prevalence of musculoskeletal complaints, this measurement method often raises concerns related to recall bias for self-reported outcomes in a 12-month period. Studies have shown that recall of disability for up to 6 months can be a reliable assessment method [43]. However, there is conflicting evidence regarding the reliability of recall beyond six months up to 12 months. The self-reporting method used in these studies also focused on the location of symptoms and the range of time of its manifestation without reporting the duration or intensity of symptoms or other assessment forms and still lacks data from physical exams and evaluations, which were recommended in a prior review with dental professionals [1]. It is widely acknowledged that meta-analyses are inherently diverse regarding clinical and methodological factors, despite using the Nordic Questionnaire or comparable ones. As a result, statistical heterogeneity is often unavoidable. The existence of a genuinely homogeneous group of studies within a meta-analysis, leading to zero heterogeneity, is considered to be impractical in real-world applications [44]. Our study found that most of the meta-analyses performed exhibited considerable heterogeneity, with I2 values ranging from 75% to 100% [44].

Despite the insights gained from this meta-analysis, it is essential to acknowledge the limitations of the available studies. The results are based on a limited number of studies, and additional research is required to validate these findings and determine the most effective strategies for preventing and managing WMSDs in dental students. Furthermore, many included studies do not specify whether the students were enrolled in clinical practice or their college years, making it difficult to generalize the results to the broader population of dental students. Additionally, the preference for cross-sectional studies (15) over prospective studies (1) and the lack of detailed descriptive statistics in some studies prevented a meta-regression analysis, limiting the depth of the insights that can be gained from this meta-analysis. Nevertheless, the high prevalence of WMSDs in this population highlights the importance of addressing this issue and the need for further research in this area. However, further investigations should have a prospective design that may allow a better understanding of this problem and its associated factors.

## 5 Conclusion

The high prevalence of work-related musculoskeletal disorders among dental students is concerning, particularly in the cervicothoracic, lumbar, and shoulder regions. Female sex, poor posture habits, inadequate ergonomics knowledge, sedentary lifestyle, high physical activity levels, poor quality of life, and smoking have been identified as contributing factors to WMSDs, and physical exercise has been found to have a protective impact over this problem. This review highlights the significant impact that WMSDs have on the dental profession, even from the training years, with a high incidence of trunk and upper extremity musculoskeletal pain being a concern for the occupational well-being of dental students. Given the limited number of studies focused on dental students and conflicting findings in current research, it is crucial to conduct more extensive prospective/longitudinal studies with larger populations and increased response rates to ensure the generalizability of the results and conclusions. A multidimensional approach to self-reported symptoms, including psychosocial constructs and physical assessments, is needed to understand better this relevant issue and its impact on the future of these students and ensure the sustainability of the dental profession.

## Author contribution statement

All authors listed have significantly contributed to the development and the writing of this article and have approved the manuscript.

Manuel Barbosa de Almeida: Rita Póvoa: Raúl Oliveira: Conceived and designed the experiments; Performed the experiments; Analyzed and interpreted the data; Wrote the paper.

Duarte Tavares: Performed the experiments. Analyzed and interpreted the data; Wrote the paper.

Paula Moleirinho Alves: Conceived and designed the experiments; Wrote the paper.

## Funding statement

Our study was developed within the scope of the Egas Moniz Multidisciplinary Research Center (CiiEM) and funded by Portugal’s national funds through the FCT – Foundation for Science and Technology, I.P. under the project UIDB/04585/2020.

## Data availability statement

The datasets used during the current study are published in open access and available from https://data.mendeley.com/datasets/grtwryxfbd/2.

## Declaration of competing interest

The authors declare that they have no known competing financial interests or personal relationships that could have appeared to influence the work reported in this paper.
