# Supplementary material for: Prevalence of musculoskeletal disorders among dental students: A systematic review and meta-analysis
Source: Heliyon. 2023 Sep 11;9(10):e19956. doi: 10.1016/j.heliyon.2023.e19956 (PMC10539954; doi:10.1016/j.heliyon.2023.e19956)
Supplement: Multimedia component 1 [file mmc1.docx]

# APPENDIX 1

**Review question**

What is the prevalence of musculoskeletal disorders among dental students and what are the contributing factors?

We will include cross-sectional studies that are conducted with dental students with prevalence of musculoskeletal disorders assessed using a standard and valid measure and don't include students and professional dentists in the same group or physically impaired subjects.

**Inclusion criteria**

Study conducted with dental students;

Prevalence of musculoskeletal disorders assessed using a standard and valid measure;

Article published in English or Portuguese in journals with peer-review.

**Exclusion Criteria**

Studies that include students and professional dentists in the same group;

Studies with physically impaired subjects;

Studies that exclude participants based on sociodemographic features;

Grey literature, letters to authors and opinion articles.

**Search strategy**

(student OR undergraduate) AND (dentist or dental) AND (musculoskeletal OR orthopedic OR orthopaedic) AND (symptom or disorder or dysfunction or injur*) AND (prevalence)

**Databases**

PubMed, COCHRANE, EBSCO and Scopus electronic databases since their inception to August 2022.

# APPENDIX 2

Work-related musculoskeletal disorders in the previous 7 days.

| **Neck – 7 days** | | | | | |
| --- | --- | --- | --- | --- | --- |
| **Author** | **Size**  (n) | **ER**  (%) | **95% CI**  [range] | **Event rate and 95% CI** | **Weight** (%) |
| Aboalshamat 2020 | 274 | 10.6 | [7.2; 14.9] | 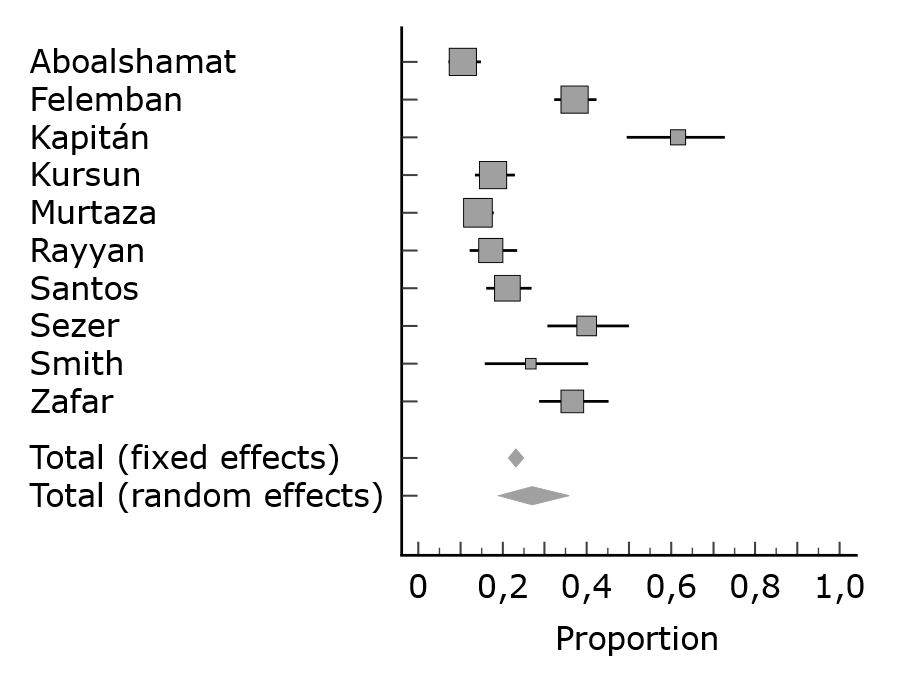 | 10.3 |
| Felemban 2021 | 377 | 37.1 | [32.2; 42.2] |  | 10.5 |
| Kapitán 2021 | 73 | 61.6 | [49.5; 72.8] |  | 9.3 |
| Kurşun 2014 | 264 | 17.8 | [13.4; 23.0] |  | 10.3 |
| Murtaza 2021 | 422 | 14.2 | [11.0; 17.9] |  | 10.5 |
| Rayyan 2016 | 191 | 17.3 | [12.2; 23.4] |  | 10.2 |
| Santos 2019 | 241 | 21.2 | [16.2; 26.9] |  | 10.3 |
| Sezer 2022 | 105 | 40.0 | [30.6; 50.0] |  | 9.7 |
| Smith 2009 | 56 | 26.8 | [15.8; 40.3] |  | 9.0 |
| Zafar 2019 | 142 | 36.6 | [28.7; 45.1] |  | 10.0 |
|  |  |  |  |  |  |
| Fixed Effects model | 2145 | 23.2 | [21.4; 25.0] |  | 100 |
| **Random effects model** | **2145** | **27.0** | **[19.1; 35.8]** |  | **100** |
| Heterogeneity: Q=172.4; p<0.0001  I^2^= 94.8%, [92.2; 96.5] | | | |  |  |

| **Shoulders – 7 days** | | | | | |
| --- | --- | --- | --- | --- | --- |
| **Author** | **Size**  (n) | **ER**  (%) | **95% CI**  [range] | **Event rate and 95% CI** | **Weight** (%) |
| Aboalshamat 2020 | 274 | 12.0 | [8.4; 16.5] | 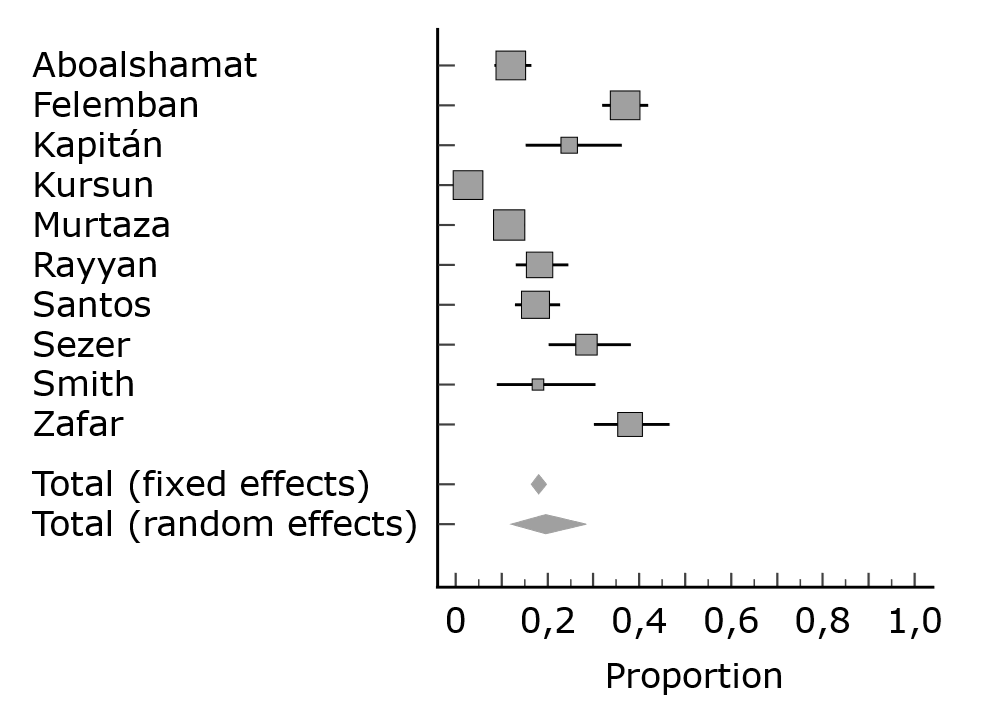 | 10.3 |
| Felemban 2021 | 377 | 36.9 | [32.0; 42.0] |  | 10.4 |
| Kapitán 2021 | 73 | 24.7 | [15.3; 36.1] |  | 9.4 |
| Kurşun 2014 | 264 | 2.7 | [1.1; 5.4] |  | 10.3 |
| Murtaza 2021 | 422 | 11.6 | [8.7; 15.1] |  | 10.4 |
| Rayyan 2016 | 191 | 18.3 | [13.1; 24.6] |  | 10.1 |
| Santos 2019 | 241 | 17.4 | [12.9; 22.8] |  | 10.2 |
| Sezer 2022 | 105 | 28.6 | [20.2; 38.2] |  | 9.8 |
| Smith 2009 | 56 | 17.9 | [8.9; 30.4] |  | 9.1 |
| Zafar 2019 | 142 | 38.0 | [30.0; 46.6] |  | 10.0 |
|  |  |  |  |  |  |
| Fixed Effects model | 2145 | 18.1 | [16.5; 19.8] |  | 100 |
| **Random effects model** | **2145** | **19.6** | **[12.1; 28.4]** |  | **100** |
| Heterogeneity: Q=206.0; p<0.0001  I^2^= 95.6%, [93.6; 97.1] | | | |  |  |

| **Elbows – 7 days** | | | | | |
| --- | --- | --- | --- | --- | --- |
| **Author** | **Size**  (n) | **ER**  (%) | **95% CI**  [range] | **Event rate and 95% CI** | **Weight** (%) |
| Aboalshamat 2020 | 274 | 14.2 | [10.3; 18.9] | 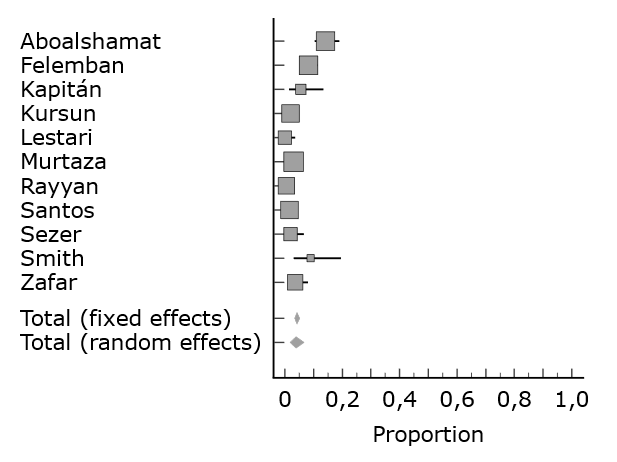 | 12.2 |
| Felemban 2021 | 377 | 8.2 | [5.7; 11.5] |  | 16.7 |
| Kapitán 2021 | 73 | 5.5 | [1.5; 13.4] |  | 3.3 |
| Kurşun 2014 | 264 | 1.9 | [0.6; 4.5] |  | 11.7 |
| Lestari 2020 | 102 | 0.0 | [0.0; 3.6] |  | 4.6 |
| Murtaza 2021 | 422 | 3.1 | [1.7; 5.2] |  | 18.7 |
| Rayyan 2016 | 191 | 0.5 | [0.0; 2.9] |  | 8.5 |
| Santos 2019 | 241 | 1.7 | [0.5; 4.2] |  | 10.7 |
| Sezer 2022 | 105 | 1.9 | [0.2; 6.7] |  | 4.7 |
| Smith 2009 | 56 | 8.9 | [3.0; 19.6] |  | 2.5 |
| Zafar 2019 | 142 | 3.5 | [1.2; 8.0] |  | 6.3 |
|  |  |  |  |  |  |
| Fixed Effects model | 2247 | 4.2 | [3.4; 5.1] |  | 100 |
| **Random effects model** | **2247** | **3.9** | **[1.9; 6.6]** |  | **100** |
| Heterogeneity: Q=83.4; p<0.0001  I^2^= 88.0%, [80.5; 92.6] | | | |  |  |

| **Hands/Wrists – 7 days** | | | | | |
| --- | --- | --- | --- | --- | --- |
| **Author** | **Size**  (n) | **ER**  (%) | **95% CI**  [range] | **Event rate and 95% CI** | **Weight** (%) |
| Aboalshamat 2020 | 274 | 23.7 | [18.8; 29.2] | 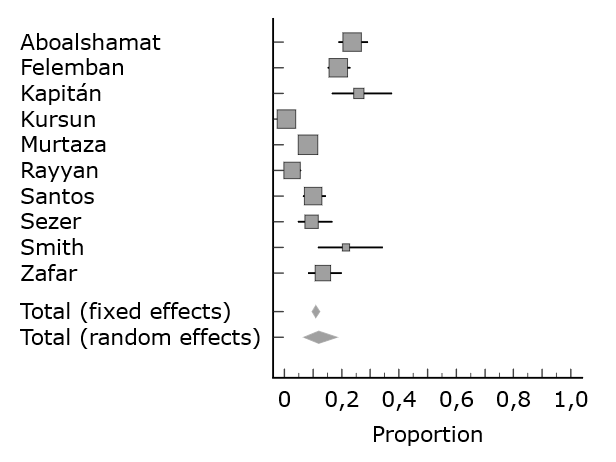 | 10.4 |
| Felemban 2021 | 377 | 18.8 | [15.0; 23.2] |  | 10.5 |
| Kapitán 2021 | 73 | 26.0 | [16.5; 37.6] |  | 9.3 |
| Kurşun 2014 | 264 | 0.8 | [0.1; 2.7] |  | 10.4 |
| Murtaza 2021 | 422 | 8.3 | [5.9; 11.4] |  | 10.5 |
| Rayyan 2016 | 191 | 2.6 | [0.9; 6.0] |  | 10.2 |
| Santos 2019 | 241 | 10.0 | [6.5; 14.5] |  | 10.3 |
| Sezer 2022 | 105 | 9.5 | [4.7; 16.8] |  | 9.7 |
| Smith 2009 | 56 | 21.4 | [11.6; 34.4] |  | 8.9 |
| Zafar 2019 | 142 | 13.4 | [8.3; 20.1] |  | 10.0 |
|  |  |  |  |  |  |
| Fixed Effects model | 2145 | 11.0 | [9.7; 12.4] |  | 100 |
| **Random effects model** | **2145** | **12.0** | **[6.8; 18.5]** |  | **100** |
| Heterogeneity: Q=156.4; p<0.0001  I^2^= 94.3%, [91.3; 96.2] | | | |  |  |

| **Upper back – 7 days** | | | | | |
| --- | --- | --- | --- | --- | --- |
| **Author** | **Size**  (n) | **ER**  (%) | **95% CI**  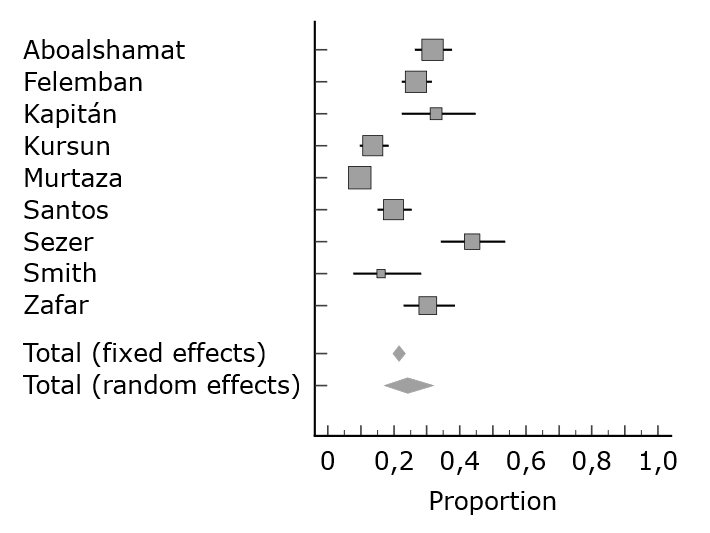[range] | **Event rate and 95% CI** | **Weight** (%) |
| Aboalshamat 2020 | 274 | 31.8 | [26.3; 37.6] |  | 11.6 |
| Felemban 2021 | 377 | 26.8 | [22.4; 31.6] |  | 11.8 |
| Kapitán 2021 | 73 | 32.9 | [22.3; 44.9] |  | 10.1 |
| Kurşun 2014 | 264 | 13.6 | [9.7; 18.4] |  | 11.6 |
| Murtaza 2021 | 422 | 9.7 | [7.1; 13.0] |  | 11.9 |
| Santos 2019 | 241 | 19.9 | [15.1; 25.5] |  | 11.6 |
| Sezer 2022 | 105 | 43.8 | [34.1; 53.8] |  | 10.7 |
| Smith 2009 | 56 | 16.1 | [7.6; 28.3] |  | 9.6 |
| Zafar 2019 | 142 | 30.3 | [22.9; 38.6] |  | 11.1 |
|  |  |  |  |  |  |
| Fixed Effects model | 1954 | 21.6 | [19.8; 23.5] |  | 100 |
| **Random effects model** | **1954** | **24.2** | **[17.2; 32.0]** |  | **100** |
| Heterogeneity: Q=114.3; p<0.0001  I^2^= 93.0%, [88.9; 95.6] | | | |  |  |

| **Lower back – 7 days** | | | | | |
| --- | --- | --- | --- | --- | --- |
| **Author** | **Size**  (n) | **ER**  (%) | **95% CI**  [range] | **Event rate and 95% CI** | **Weight** (%) |
| Aboalshamat 2020 | 274 | 13.9 | [10.0; 18.5] | 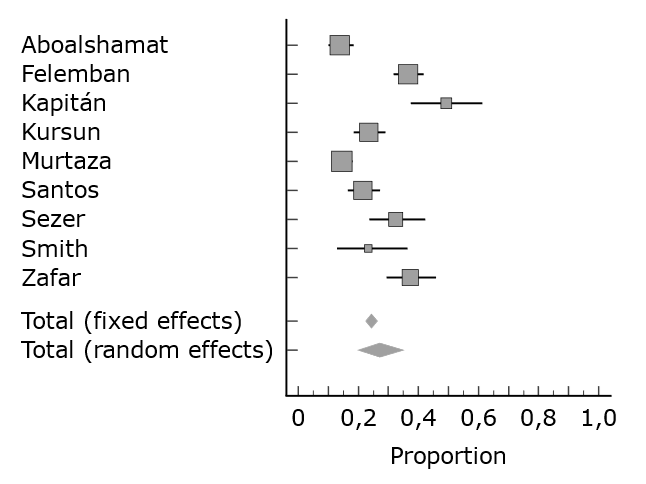 | 11.7 |
| Felemban 2021 | 377 | 36.6 | [31.7; 41.7] |  | 11.8 |
| Kapitán 2021 | 73 | 49.3 | [37.4; 61.3] |  | 10.1 |
| Kurşun 2014 | 264 | 23.5 | [18.5; 29.1] |  | 11.6 |
| Murtaza 2021 | 422 | 14.5 | [11.2; 18.2] |  | 11.9 |
| Santos 2019 | 241 | 21.6 | [16.6; 27.3] |  | 11.6 |
| Sezer 2022 | 105 | 32.4 | [23.6; 42.2] |  | 10.7 |
| Smith 2009 | 56 | 23.2 | [13.0; 36.4] |  | 9.6 |
| Zafar 2019 | 142 | 37.3 | [29.4; 45.8] |  | 11.1 |
|  |  |  |  |  |  |
| Fixed Effects model | 1954 | 24.4 | [22.5; 26.3] |  | 100 |
| **Random effects model** | **1954** | **27.2** | **[20.0; 35.0]** |  | **100** |
| Heterogeneity: Q=109.4; p<0.0001  I^2^= 92.7%, [88.3; 95.4] | | | |  |  |

| **Hips/Thighs – 7 days** | | | | | |
| --- | --- | --- | --- | --- | --- |
| **Author** | **Size**  (n) | **ER**  (%) | **95% CI**  [range] | **Event rate and 95% CI** | **Weight** (%) |
| Aboalshamat 2020 | 274 | 17.5 | [13.2; 22.6] | 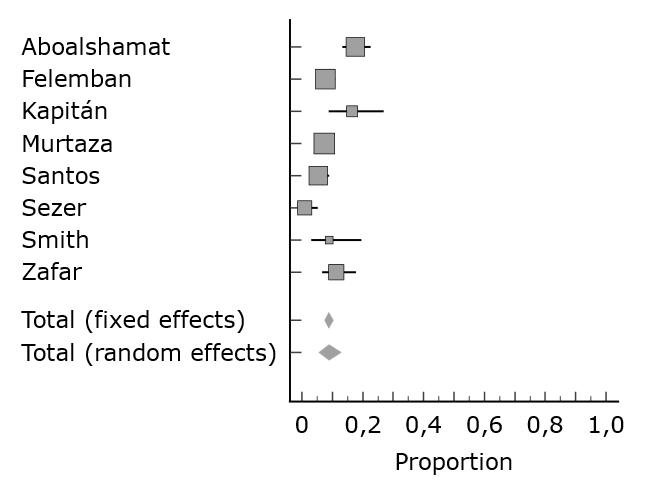 | 13.8 |
| Felemban 2021 | 377 | 7.7 | [5.2; 10.9] |  | 14.3 |
| Kapitán 2021 | 73 | 16.4 | [8.8; 27.0] |  | 10.4 |
| Murtaza 2021 | 422 | 7.3 | [5.1; 10.3] |  | 14.4 |
| Santos 2019 | 241 | 5.4 | [2.9; 9.1] |  | 13.6 |
| Sezer 2022 | 105 | 1.0 | [0.0; 5.2] |  | 11.6 |
| Smith 2009 | 56 | 8.9 | [3.0; 19.6] |  | 9.5 |
| Zafar 2019 | 142 | 11.3 | [6.6; 17.7] |  | 12.4 |
|  |  |  |  |  |  |
|  |  |  |  |  |  |
| Fixed Effects model | 1690 | 8.8 | [7.5; 10.3] |  | 100 |
| **Random effects model** | **1690** | **8.9** | **[5.6; 12.9]** |  | **100** |
| Heterogeneity: Q=44.5; p<0.0001  I^2^= 84.3%, [70.7; 91.6] | | | |  |  |

| **Knees – 7 days** | | | | | |
| --- | --- | --- | --- | --- | --- |
| **Author** | **Size**  (n) | **ER**  (%) | **95% CI**  [range] | **Event rate and 95% CI** | **Weight** (%) |
| Aboalshamat 2020 | 274 | 19.7 | [15.2; 24.9] | 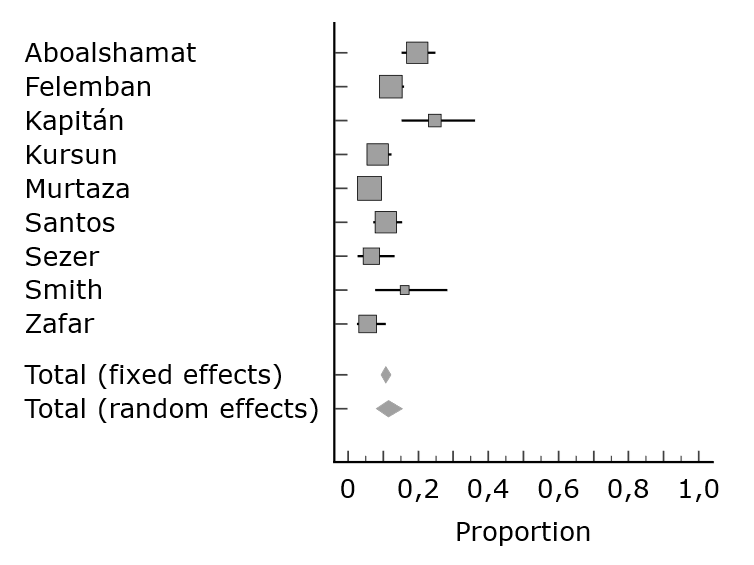 | 12.2 |
| Felemban 2021 | 377 | 12.2 | [9.1; 15.9] |  | 12.6 |
| Kapitán 2021 | 73 | 24.7 | [15.3; 36.1] |  | 9.1 |
| Kurşun 2014 | 264 | 8.3 | [5.3; 12.4] |  | 12.1 |
| Murtaza 2021 | 422 | 6.2 | [4.1; 8.9] |  | 12.8 |
| Santos 2019 | 241 | 10.8 | [7.2; 15.4] |  | 12.0 |
| Sezer 2022 | 105 | 6.7 | [2.7; 13.3] |  | 10.1 |
| Smith 2009 | 56 | 16.1 | [7.6; 28.3] |  | 8.2 |
| Zafar 2019 | 142 | 5.6 | [2.5; 10.8] |  | 10.9 |
|  |  |  |  |  |  |
| Fixed Effects model | 1954 | 10.8 | [9.4; 12.2] |  | 100 |
| **Random effects model** | **1954** | **11.5** | **[8.1; 15.5]** |  | **100** |
| Heterogeneity: Q=50.0; p<0.0001  I^2^= 84.0%, [71.2; 91.1] | | | |  |  |

| **Ankles/Feet – 7 days** | | | | | |
| --- | --- | --- | --- | --- | --- |
| **Author** | **Size**  (n) | **ER**  (%) | **95% CI**  [range] | **Event rate and 95% CI** | **Weight** (%) |
| Aboalshamat 2020 | 274 | 15.3 | [11.3; 20.2] | 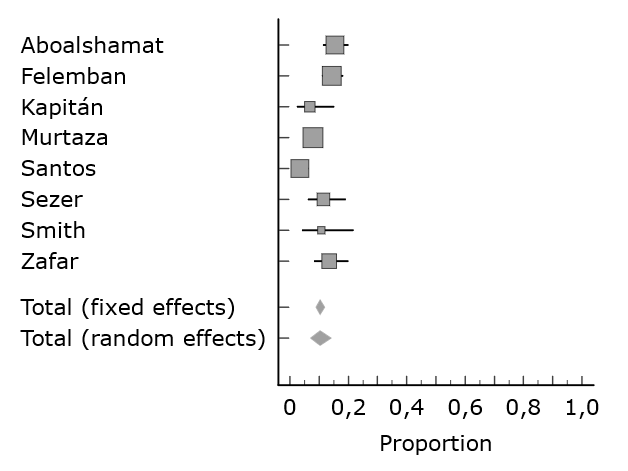 | 14.1 |
| Felemban 2021 | 377 | 14.3 | [11.0; 18.3] |  | 14.6 |
| Kapitán 2021 | 73 | 6.8 | [2.3; 15.3] |  | 10.0 |
| Murtaza 2021 | 422 | 7.8 | [5.4; 10.8] |  | 14.8 |
| Santos 2019 | 241 | 3.3 | [1.4; 6.4] |  | 13.8 |
| Sezer 2022 | 105 | 11.4 | [6.1; 19.1] |  | 11.4 |
| Smith 2009 | 56 | 10.7 | [4.0; 21.9] |  | 9.0 |
| Zafar 2019 | 142 | 13.4 | [8.3; 20.1] |  | 12.4 |
|  |  |  |  |  |  |
| Fixed Effects model | 1690 | 10.3 | [8.9; 11.9] |  | 100 |
| **Random effects model** | **1690** | **10.3** | **[7.1; 14.1]** |  | **100** |
| Heterogeneity: Q=36.5; p<0.0001  I^2^= 80.8%, [63.1; 90.0] | | | |  |  |

Work-related musculoskeletal disorders in the previous 12 months.

| **Neck – 12 months** | | | | | |
| --- | --- | --- | --- | --- | --- |
| **Author** | **Size**  (n) | **ER**  (%) | **95% CI**  [range] | **Event rate and 95% CI** | **Weight** (%) |
| Aboalshamat 2020 | 274 | 38.3 | [32.5; 44.4] | 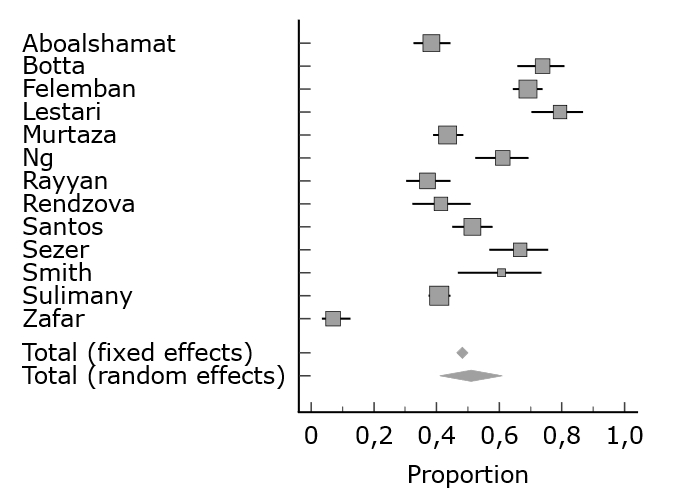 | 7.9 |
| Botta 2018 | 145 | 73.8 | [65.9; 80.7] |  | 7.7 |
| Felemban 2021 | 377 | 69.2 | [64.3 73.9] |  | 7.9 |
| Lestari 2020 | 102 | 79.4 | [70.3; 86.8] |  | 7.5 |
| Murtaza 2021 | 422 | 43.6 | [38.8; 48.5] |  | 7.9 |
| Ng 2016 | 136 | 61.0 | [52.3; 69.3] |  | 7.7 |
| Rayyan 2016 | 191 | 37.2 | [30.3; 44.5] |  | 7.8 |
| Rendzova 2021 | 116 | 41.4 | [32.3; 50.9] |  | 7.6 |
| Santos 2019 | 241 | 51.5 | [45.0; 57.9] |  | 7.8 |
| Sezer 2022 | 105 | 66.7 | [56.8; 75.6] |  | 7.5 |
| Smith 2009 | 56 | 60.7 | [46.8; 73.5] |  | 7.1 |
| Sulimany 2021 | 794 | 40.9 | [37.5; 44.4] |  | 8.0 |
| Zafar 2019 | 142 | 7.0 | [3.4; 12.6] |  | 7.7 |
|  |  |  |  |  |  |
| Fixed Effects model | 3101 | 48.2 | [46.5; 50.0] |  | 100 |
| **Random effects model** | **3101** | **51.0** | **[41.0; 61.0]** |  | **100** |
| Heterogeneity: Q=365.5; p<0.0001  I^2^= 96.7%, [95.6; 97.6] | | | |  |  |

| **Shoulders – 12 months** | | | | | |
| --- | --- | --- | --- | --- | --- |
| **Author** | **Size**  (n) | **ER**  (%) | **95% CI**  [range] | **Event rate and 95% CI** | **Weight** (%) |
| Aboalshamat 2020 | 274 | 33.6 | [28.0; 39.5] | 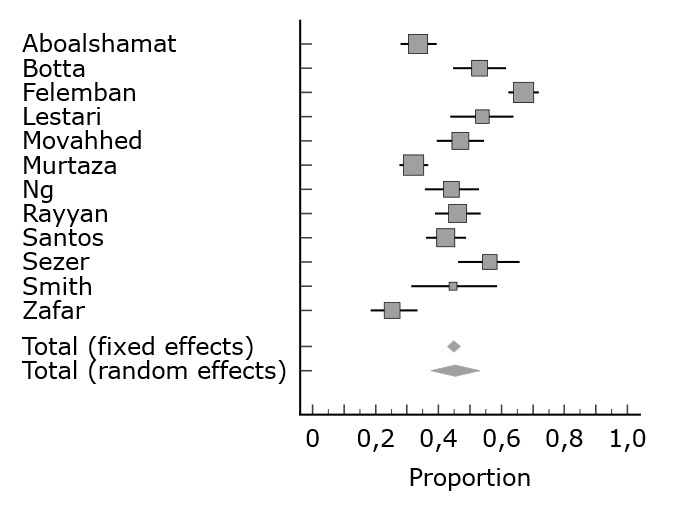 | 8.7 |
| Botta 2018 | 145 | 53.1 | [44.7; 61.4] |  | 8.3 |
| Felemban 2021 | 377 | 67.1 | [62.1; 71.8] |  | 8.8 |
| Lestari 2020 | 102 | 53.9 | [43.8; 63.8] |  | 8.0 |
| Movahhed 2013 | 177 | 46.9 | [39.4; 54.5] |  | 8.4 |
| Murtaza 2021 | 422 | 32.0 | [27.6; 36.7] |  | 8.8 |
| Ng 2016 | 136 | 44.1 | [35.6; 52.9] |  | 8.3 |
| Rayyan 2016 | 191 | 46.1 | [38.9; 53.4] |  | 8.5 |
| Santos 2019 | 241 | 42.3 | [36.0; 48.8] |  | 8.6 |
| Sezer 2022 | 105 | 56.2 | [46.2; 65.9] |  | 8.0 |
| Smith 2009 | 56 | 44.6 | [31.3; 58.5] |  | 7.3 |
| Zafar 2019 | 142 | 25.4 | [18.4; 33.3] |  | 8.3 |
|  |  |  |  |  |  |
| Fixed Effects model | 2368 | 44.9 | [42.9; 46.9] |  | 100 |
| **Random effects model** | **2368** | **45.3** | **[37.6; 53.1]** |  | **100** |
| Heterogeneity: Q=158.9; p<0.0001  I^2^= 93.1%, [89.7; 95.3] | | | |  |  |

| **Elbows – 12 months** | | | | | |
| --- | --- | --- | --- | --- | --- |
| **Author** | **Size**  (n) | **ER**  (%) | **95% CI**  [range] | **Event rate and 95% CI** | **Weight** (%) |
| Aboalshamat 2020 | 274 | 31.8 | [26.3; 37.6] | 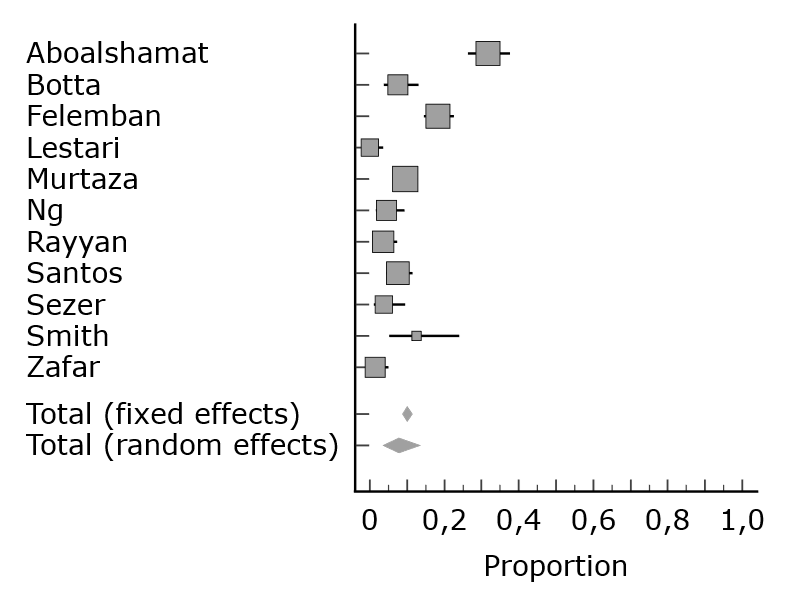 | 9.4 |
| Botta 2018 | 145 | 7.6 | [3.9; 13.2] |  | 9.1 |
| Felemban 2021 | 377 | 18.3 | [14.; 22.6] |  | 9.5 |
| Lestari 2020 | 102 | 0.0 | [0.0; 3.6] |  | 8.8 |
| Murtaza 2021 | 422 | 9.5 | [6.9; 12.7] |  | 9.5 |
| Ng 2016 | 136 | 4.4 | [1.6; 9.4] |  | 9.0 |
| Rayyan 2016 | 191 | 3.7 | [1.5; 7.4] |  | 9.2 |
| Santos 2019 | 241 | 7.5 | [4.5; 11.6] |  | 9.3 |
| Sezer 2022 | 105 | 3.8 | [1.1; 9.5] |  | 8.8 |
| Smith 2009 | 56 | 12.5 | [5.2; 24.1] |  | 8.2 |
| Zafar 2019 | 142 | 1.4 | [0.2; 5.0] |  | 9.1 |
|  |  |  |  |  |  |
| Fixed Effects model | 2191 | 10.0 | [8.8; 11.4] |  | 100 |
| **Random effects model** | **2191** | **7.8** | **[3.6; 13.4]** |  | **100** |
| Heterogeneity: Q=184.2; p<0.0001  I^2^= 94.6%, [92.0; 96.3] | | | |  |  |

| **Hands/Wrists – 12 months** | | | | | |
| --- | --- | --- | --- | --- | --- |
| **Author** | **Size**  (n) | **ER**  (%) | **95% CI**  [range] | **Event rate and 95% CI** | **Weight** (%) |
| Aboalshamat 2020 | 274 | 48.5 | [42.5; 54.6] | 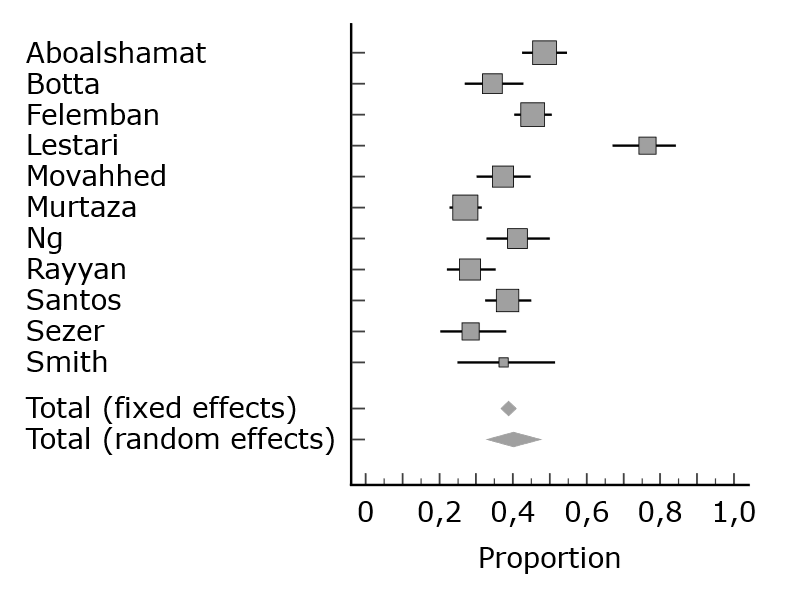 | 9.5 |
| Botta 2018 | 145 | 34.5 | [26.8; 42.8] |  | 9.0 |
| Felemban 2021 | 377 | 45.4 | [40.3; 50.5] |  | 9.7 |
| Lestari 2020 | 102 | 76.5 | [67.0; 84.3] |  | 8.7 |
| Movahhed 2013 | 177 | 37.3 | [30.2; 44.9] |  | 9.2 |
| Murtaza 2021 | 422 | 27.0 | [22.8; 31.5] |  | 9.7 |
| Ng 2016 | 136 | 41.2 | [32.8; 49.9] |  | 9.0 |
| Rayyan 2016 | 191 | 28.3 | [22.0; 35.2] |  | 9.3 |
| Santos 2019 | 241 | 38.6 | [32.4; 45.1] |  | 9.5 |
| Sezer 2022 | 105 | 28.6 | [20.2; 38.2] |  | 8.7 |
| Smith 2009 | 56 | 37.5 | [24.9; 51.5] |  | 7.7 |
|  |  |  |  |  |  |
| Fixed Effects model | 2226 | 38.8 | [36.8; 40.9] |  | 100 |
| **Random effects model** | **2226** | **40.1** | **[33.0; 47.5]** |  | **100** |
| Heterogeneity: Q=122.0; p<0.0001  I^2^= 91.8%, [87.3; 94.7] | | | |  |  |

| **Upper back – 12 months** | | | | | |
| --- | --- | --- | --- | --- | --- |
| **Author** | **Size**  (n) | **ER**  (%) | **95% CI**  [range] | **Event rate and 95% CI** | **Weight** (%) |
| Aboalshamat 2020 | 274 | 43.8 | [37.8; 49.9] | 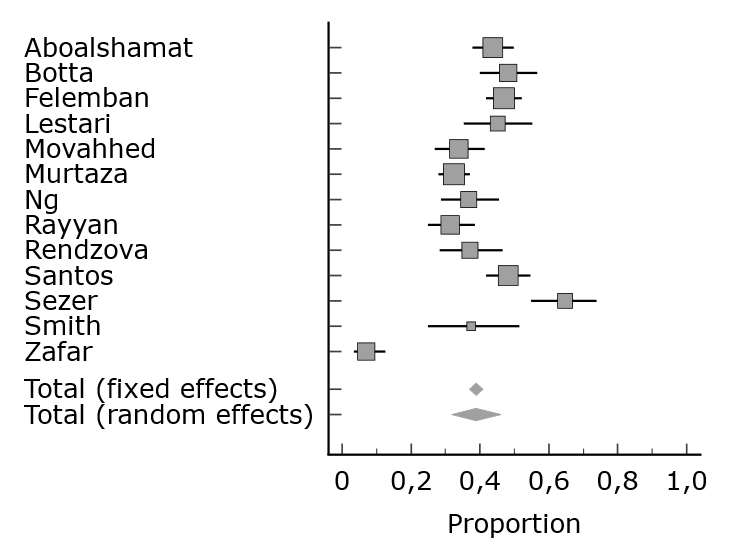 | 8.0 |
| Botta 2018 | 145 | 48.3 | [39.9; 56.7] |  | 7.7 |
| Felemban 2021 | 377 | 47.0 | [41.8; 52.1] |  | 8.1 |
| Lestari 2020 | 102 | 45.1 | [35.2; 55.3] |  | 7.4 |
| Movahhed 2013 | 177 | 33.9 | [27.0; 41.4] |  | 7.8 |
| Murtaza 2021 | 422 | 32.5 | [28.0; 37.2] |  | 8.2 |
| Ng 2016 | 136 | 36.8 | [28.7; 45.5] |  | 7.6 |
| Rayyan 2016 | 191 | 31.4 | [24.9; 38.5] |  | 7.9 |
| Rendzova 2021 | 116 | 37.1 | [28.3; 46.5] |  | 7.5 |
| Santos 2019 | 241 | 48.1 | [41.7; 54.6] |  | 8.0 |
| Sezer 2022 | 105 | 64.8 | [54.8; 73.8] |  | 7.4 |
| Smith 2009 | 56 | 37.5 | [24.9; 51.5] |  | 6.7 |
| Zafar 2019 | 142 | 7.0 | [3.4; 12.6] |  | 7.7 |
|  |  |  |  |  |  |
| Fixed Effects model | 2484 | 38.9 | [37.0; 40.9] |  | 100 |
| **Random effects model** | **2484** | **38.9** | **[31.8; 46.1]** |  | **100** |
| Heterogeneity: Q=162.4; p<0.0001  I^2^= 92.6%, [89.1; 95.0] | | | |  |  |

| **Lower back – 12 months** | | | | | |
| --- | --- | --- | --- | --- | --- |
| **Author** | **Size**  (n) | **ER**  (%) | **95% CI**  [range] | **Event rate and 95% CI** | **Weight** (%) |
| Aboalshamat 2020 | 274 | 45.6 | [39.6; 51.7] | 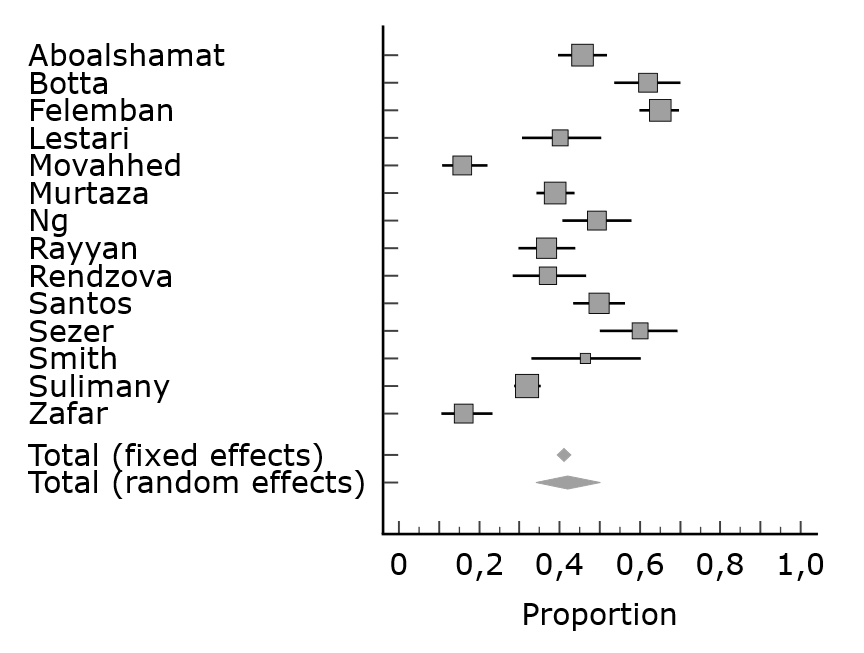 | 7.4 |
| Botta 2018 | 145 | 62.1 | [53.7; 70.0] |  | 7.1 |
| Felemban 2021 | 377 | 65.0 | [59.9; 69.8] |  | 7.4 |
| Lestari 2020 | 102 | 40.2 | [30.6; 50.4] |  | 6.9 |
| Movahhed 2013 | 177 | 15.8 | [10.8; 22.1] |  | 7.2 |
| Murtaza 2021 | 422 | 38.9 | [34.2; 43.7] |  | 7.5 |
| Ng 2016 | 136 | 49.3 | [40.6; 58.0] |  | 7.1 |
| Rayyan 2016 | 191 | 36.6 | [29.8; 43.9] |  | 7.2 |
| Rendzova 2021 | 116 | 37.1 | [28.3; 46.5] |  | 7.0 |
| Santos 2019 | 241 | 49.8 | [43.3; 56.3] |  | 7.3 |
| Sezer 2022 | 105 | 60.0 | [50.0; 69.4] |  | 6.9 |
| Smith 2009 | 56 | 46.4 | [33.0; 60.3] |  | 6.4 |
| Sulimany 2021 | 794 | 31.9 | [28.6; 35.2] |  | 7.5 |
| Zafar 2019 | 142 | 16.2 | [10.6; 23.3] |  | 7.1 |
|  |  |  |  |  |  |
| Fixed Effects model | 3278 | 41.1 | [39.4; 42.8] |  | 100 |
| **Random effects model** | **3278** | **42.0** | **[34.1; 50.2]** |  | **100** |
| Heterogeneity: Q=277.5; p<0.0001  I^2^= 95.3%, [93.5; 96.6] | | | |  |  |

| **Hips/Thighs – 12 months** | | | | | |
| --- | --- | --- | --- | --- | --- |
| **Author** | **Size**  (n) | **ER**  (%) | **95% CI**  [range] | **Event rate and 95% CI** | **Weight** (%) |
| Aboalshamat 2020 | 274 | 25.2 | [20.2; 30.8] | 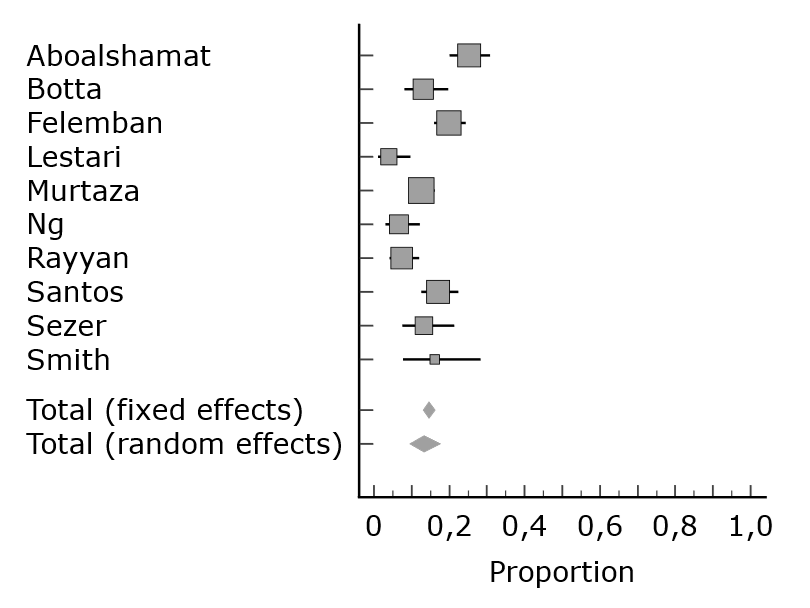 | 10.8 |
| Botta 2018 | 145 | 13.1 | [8.1; 19.7] |  | 9.9 |
| Felemban 2021 | 377 | 19.9 | [16.0; 24.3] |  | 11.2 |
| Lestari 2020 | 102 | 3.9 | [1.1; 9.7] |  | 9.2 |
| Murtaza 2021 | 422 | 12.6 | [9.6; 16.1] |  | 11.3 |
| Ng 2016 | 136 | 6.6 | [3.1; 12.2] |  | 9.8 |
| Rayyan 2016 | 191 | 7.3 | [4.1; 12.0] |  | 10.4 |
| Santos 2019 | 241 | 17.0 | [12.5; 22.4] |  | 10.7 |
| Sezer 2022 | 105 | 13.3 | [7.5; 21.4] |  | 9.2 |
| Smith 2009 | 56 | 16.1 | [7.6; 28.3] |  | 7.7 |
|  |  |  |  |  |  |
| Fixed Effects model | 2049 | 14.6 | [13.1; 16.2] |  | 100 |
| **Random effects model** | **2049** | **13.3** | **[9.6; 17.6]** |  | **100** |
| Heterogeneity: Q=62.8; p<0.0001  I^2^= 85.7%, [75.5; 91.6] | | | |  |  |

| **Knees – 12 months** | | | | | |
| --- | --- | --- | --- | --- | --- |
| **Author** | **Size**  (n) | **ER**  (%) | **95% CI**  [range] | **Event rate and 95% CI** | **Weight** (%) |
| Aboalshamat 2020 | 274 | 18.6 | [14.2; 23.7] | 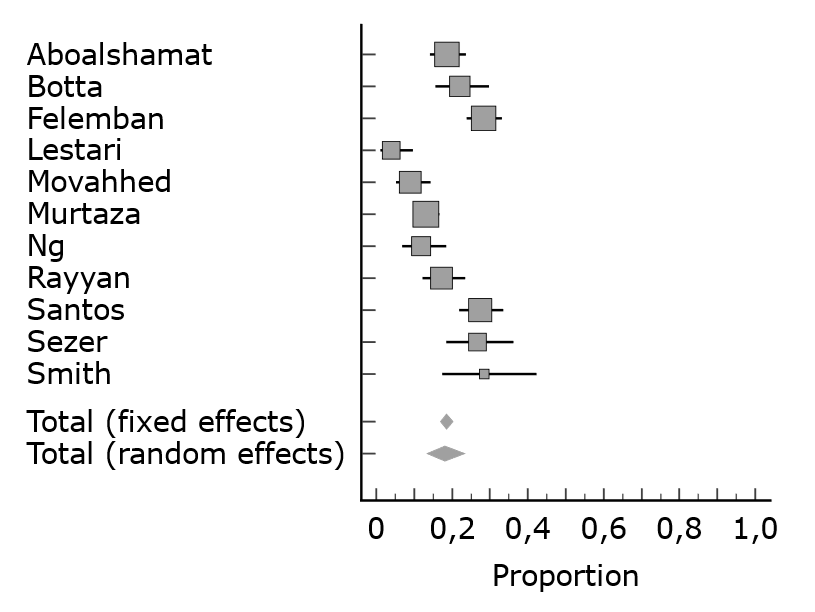 | 9.7 |
| Botta 2018 | 145 | 22.1 | [15.6; 29.7] |  | 9.0 |
| Felemban 2021 | 377 | 28.4 | [23.9; 33.2] |  | 9.9 |
| Lestari 2020 | 102 | 3.9 | [1.1; 9.7] |  | 8.5 |
| Movahhed 2013 | 177 | 9.0 | [5.3; 14.3] |  | 9.3 |
| Murtaza 2021 | 422 | 13.0 | [10.0; 16.6] |  | 10.0 |
| Ng 2016 | 136 | 11.8 | [6.9; 18.4] |  | 8.9 |
| Rayyan 2016 | 191 | 17.3 | [12.2; 23.4] |  | 9.3 |
| Santos 2019 | 241 | 27.4 | [21.9; 33.5] |  | 9.6 |
| Sezer 2022 | 105 | 26.7 | [18.5; 36.2] |  | 8.6 |
| Smith 2009 | 56 | 28.6 | [17.3; 42.2] |  | 7.4 |
|  |  |  |  |  |  |
| Fixed Effects model | 2226 | 18.5 | [17.0; 20.2] |  | 100 |
| **Random effects model** | **2226** | **18.1** | **[13.4; 23.3]** |  | **100** |
| Heterogeneity: Q=90.7; p<0.0001  I^2^= 89.0%, [82.3; 93.1] | | | |  |  |

| **Ankles/Feet – 12 months** | | | | | |
| --- | --- | --- | --- | --- | --- |
| **Author** | **Size**  (n) | **ER**  (%) | **95% CI**  [range] | **Event rate and 95% CI** | **Weight** (%) |
| Aboalshamat 2020 | 274 | 20.4 | [15.8; 25.7] | 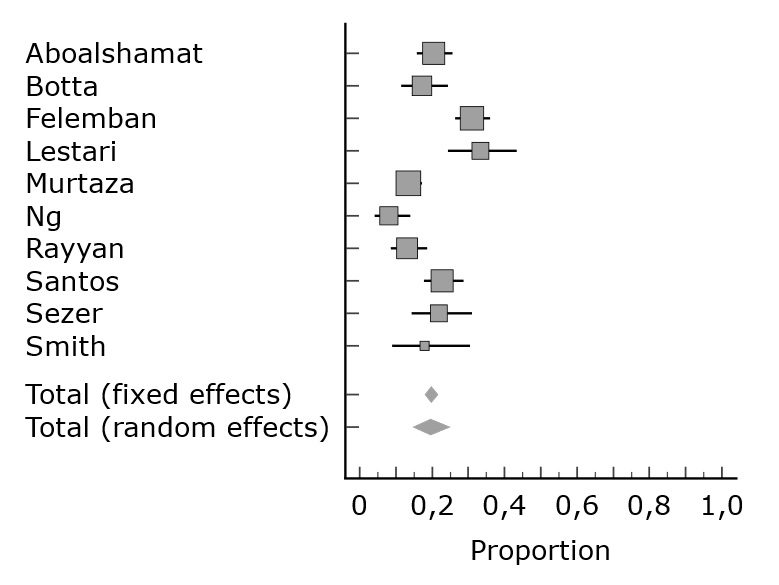 | 10.7 |
| Botta 2018 | 145 | 17.2 | [11.5; 24.4] |  | 9.9 |
| Felemban 2021 | 377 | 31.0 | [26.4; 36.0] |  | 11.0 |
| Lestari 2020 | 102 | 33.3 | [24.3; 43.4] |  | 9.3 |
| Murtaza 2021 | 422 | 13.5 | [10.4; 17.1] |  | 11.1 |
| Ng 2016 | 136 | 8.1 | [4.1; 14.0] |  | 9.8 |
| Rayyan 2016 | 191 | 13.1 | [8.7; 18.7] |  | 10.3 |
| Santos 2019 | 241 | 22.8 | [17.7; 28.7] |  | 10.6 |
| Sezer 2022 | 105 | 21.9 | [14.4; 31.0] |  | 9.3 |
| Smith 2009 | 56 | 17.9 | [8.9; 30.4] |  | 7.9 |
|  |  |  |  |  |  |
| Fixed Effects model | 2049 | 19.8 | [18.1; 21.6] |  | 100 |
| **Random effects model** | **2049** | **19.6** | **[14.9; 24.8]** |  | **100** |
| Heterogeneity: Q=71.3; p<0.0001  I^2^= 87.4%, [78.8; 92.5] | | | |  |  |

Work-related musculoskeletal disorders in the previous 12 months affecting work or normal activities.

| **Neck – 12 months, affecting work and normal activity** | | | | | |
| --- | --- | --- | --- | --- | --- |
| **Author** | **Size**  (n) | **ER**  (%) | **95% CI**  [range] | **Event rate and 95% CI** | **Weight** (%) |
| Aboalshamat 2020 | 274 | 13.1 | [9.4; 17.7] | 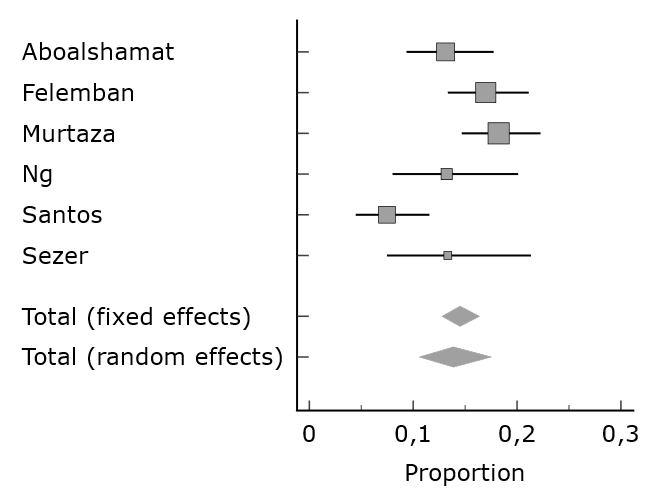 | 17.7 |
| Felemban 2021 | 377 | 17.0 | [13.3; 21.2] |  | 19.0 |
| Murtaza 2021 | 422 | 18.2 | [14.7; 22.3] |  | 19.4 |
| Ng 2016 | 136 | 13.2 | [8.0; 20.1] |  | 14.2 |
| Santos 2019 | 241 | 7.5 | [4.5; 11.6] |  | 17.1 |
| Sezer 2022 | 105 | 13.3 | [7.5; 21.4] |  | 12.7 |
|  |  |  |  |  |  |
| Fixed Effects model | 1555 | 14.5 | [12.8; 16.4] |  | 100 |
| **Random effects model** | **1555** | **13.9** | **[10.6; 17.5]** |  | **100** |
| Heterogeneity: Q=18.7; p=0.002  I^2^= 73.3%, [38.8; 88.4] | | | |  |  |

| **Shoulders – 12 months, affecting work and normal activity** | | | | | |
| --- | --- | --- | --- | --- | --- |
| **Author** | **Size**  (n) | **ER**  (%) | **95% CI**  [range] | **Event rate and 95% CI** | **Weight** (%) |
| Aboalshamat 2020 | 274 | 13.9 | [10.0; 18.5] | 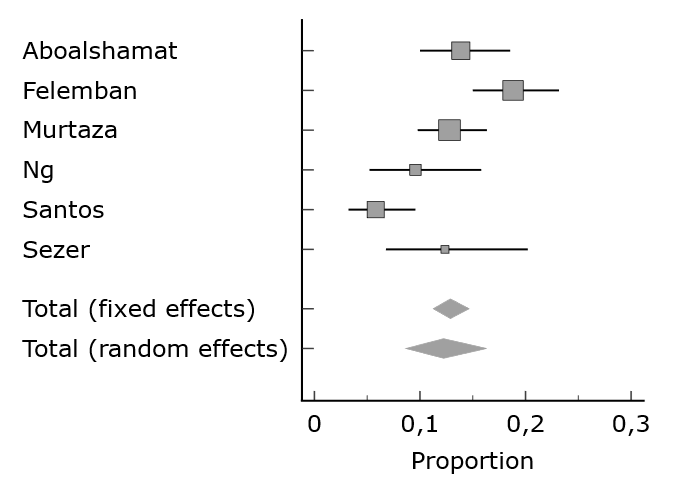 | 17.5 |
| Felemban 2021 | 377 | 18.8 | [15.0; 23.2] |  | 18.4 |
| Murtaza 2021 | 422 | 12.8 | [9.8; 16.4] |  | 18.7 |
| Ng 2016 | 136 | 9.6 | [5.2; 15.8] |  | 14.8 |
| Santos 2019 | 241 | 5.8 | [3.2; 9.6] |  | 17.1 |
| Sezer 2022 | 105 | 12.4 | [6.8; 20.2] |  | 13.6 |
|  |  |  |  |  |  |
| Fixed Effects model | 1555 | 12.9 | [11.3; 14.7] |  | 100 |
| **Random effects model** | **1555** | **12.2** | **[8.7; 16.3]** |  | **100** |
| Heterogeneity: Q=25.8; p=0.0001  I^2^= 80.6%, [58.3; 91.0] | | | |  |  |

| **Elbows – 12 months, affecting work and normal activity** | | | | | |
| --- | --- | --- | --- | --- | --- |
| **Author** | **Size**  (n) | **ER**  (%) | **95% CI**  [range] | **Event rate and 95% CI** | **Weight** (%) |
| Felemban 2021 | 377 | 10.1 | [7.2; 13.6] | 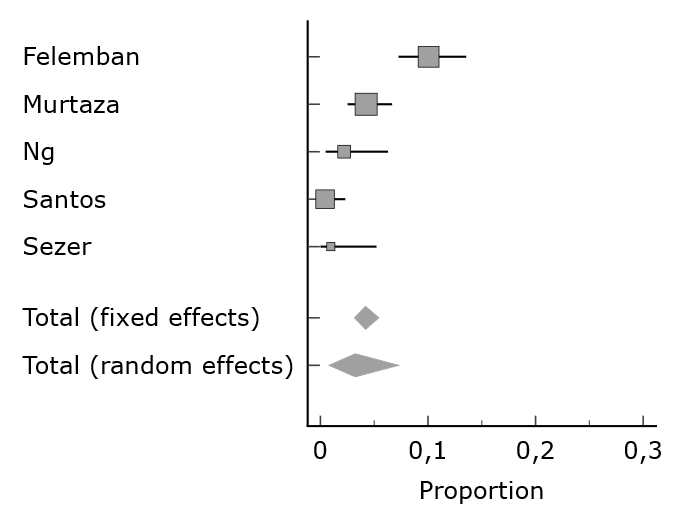 | 21.1 |
| Murtaza 2021 | 422 | 4.3 | [2.6; 6.7] |  | 21.3 |
| Ng 2016 | 136 | 2.2 | [0.5; 6.3] |  | 19.0 |
| Santos 2019 | 241 | 0.4 | [0.0; 2.3] |  | 20.4 |
| Sezer 2022 | 105 | 1.0 | [0.02; 5.2] |  | 18.2 |
|  |  |  |  |  |  |
| Fixed Effects model | 1281 | 4.2 | [3.2; 5.4] |  | 100 |
| **Random effects model** | **1281** | **3.2** | **[0.8; 7.3]** |  | **100** |
| Heterogeneity: Q=41.8; p<0.0001  I^2^= 90.4%, [80.6; 95.3] | | | |  |  |

| **Hands/Wrists – 12 months, affecting work and normal activity** | | | | | |
| --- | --- | --- | --- | --- | --- |
| **Author** | **Size**  (n) | **ER**  (%) | **95% CI**  [range] | **Event rate and 95% CI** | **Weight** (%) |
| Felemban 2021 | 377 | 15.7 | [12.1; 19.7] | 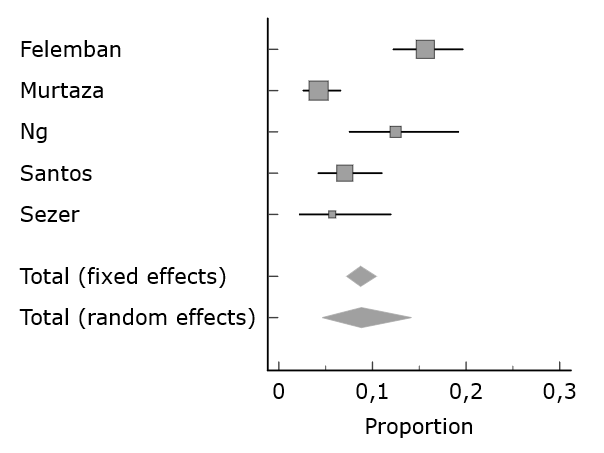 | 21.3 |
| Murtaza 2021 | 422 | 4.3 | [2.6; 6.7] |  | 21.5 |
| Ng 2016 | 136 | 12.5 | [7.5; 19.3] |  | 18.8 |
| Santos 2019 | 241 | 7.1 | [4.2; 11.1] |  | 20.5 |
| Sezer 2022 | 105 | 5.7 | [2.1; 12.0] |  | 17.9 |
|  |  |  |  |  |  |
| Fixed Effects model | 1281 | 8.7 | [7.3; 10.4] |  | 100 |
| **Random effects model** | **1281** | **8.8** | **[4.6; 14.2]** |  | **100** |
| Heterogeneity: Q=35.3; p<0.0001  I^2^= 88.7%, [76.2; 94.6] | | | |  |  |

| **Upper back – 12 months, affecting work and normal activity** | | | | | |
| --- | --- | --- | --- | --- | --- |
| **Author** | **Size**  (n) | **ER**  (%) | **95% CI**  [range] | **Event rate and 95% CI** | **Weight** (%) |
| Felemban 2021 | 377 | 14.3 | [11.0; 18.3] | 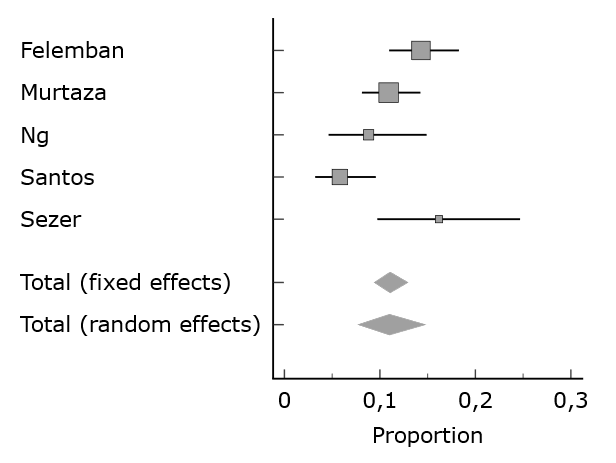 | 23.0 |
| Murtaza 2021 | 422 | 10.9 | [8.1; 14.3] |  | 23.4 |
| Ng 2016 | 136 | 8.8 | [4.6; 14.9] |  | 17.3 |
| Santos 2019 | 241 | 5.8 | [3.2; 9.6] |  | 20.8 |
| Sezer 2022 | 105 | 16.2 | [9.7; 24.7] |  | 15.6 |
|  |  |  |  |  |  |
| Fixed Effects model | 1281 | 11.1 | [9.4; 12.9] |  | 100 |
| **Random effects model** | **1281** | **11.0** | **[7.7; 14.8]** |  | **100** |
| Heterogeneity: Q=15.2; p=0.004  I^2^= 73.8%, [34.7; 89.5] | | | |  |  |

| **Lower back – 12 months, affecting work and normal activity** | | | | | |
| --- | --- | --- | --- | --- | --- |
| **Author** | **Size**  (n) | **ER**  (%) | **95% CI**  [range] | **Event rate and 95% CI** | **Weight** (%) |
| Aboalshamat 2020 | 274 | 14.6 | [10.6; 19.3] | 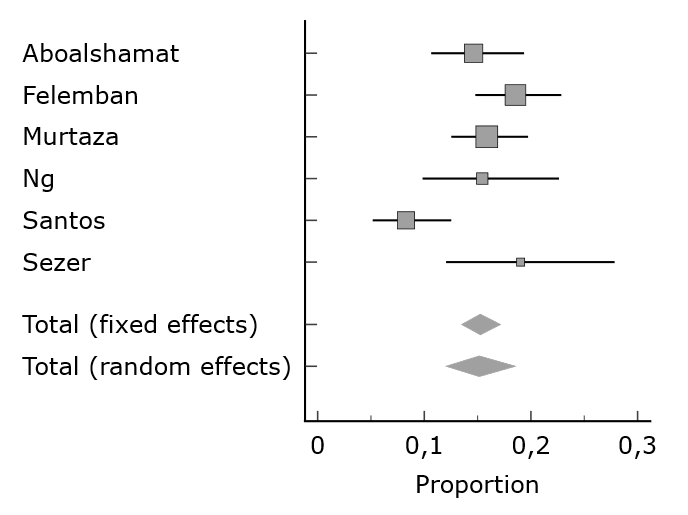 | 17.8 |
| Felemban 2021 | 377 | 18.6 | [14.8; 22.9] |  | 19.4 |
| Murtaza 2021 | 422 | 15.9 | [12.5; 19.7] |  | 19.9 |
| Ng 2016 | 136 | 15.4 | [9.8; 22.6] |  | 13.7 |
| Santos 2019 | 241 | 8.3 | [5.1; 12.5] |  | 17.1 |
| Sezer 2022 | 105 | 19.0 | [12.0; 27.9] |  | 12.0 |
|  |  |  |  |  |  |
| Fixed Effects model | 1555 | 15.3 | [13.5; 17.1] |  | 100 |
| **Random effects model** | **1555** | **15.2** | **[12.1; 18.5]** |  | **100** |
| Heterogeneity: Q=15.5; p=0.009  I^2^= 67.6%, [23.2; 86.4] | | | |  |  |

| **Hips/Thighs – 12 months, affecting work and normal activity** | | | | | |
| --- | --- | --- | --- | --- | --- |
| **Author** | **Size**  (n) | **ER**  (%) | **95% CI**  [range] | **Event rate and 95% CI** | **Weight** (%) |
| Felemban 2021 | 377 | 6.9 | [4.6; 10.0] | 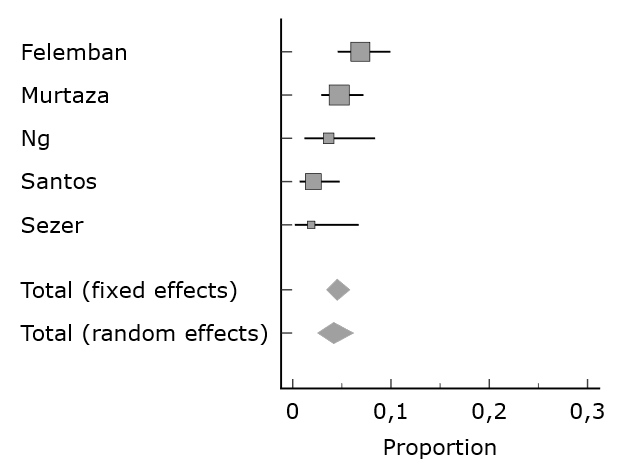 | 24.3 |
| Murtaza 2021 | 422 | 4.7 | [2.9; 7.2] |  | 25.1 |
| Ng 2016 | 136 | 3.7 | [1.2; 8.4] |  | 15.9 |
| Santos 2019 | 241 | 2.1 | [0.7; 4.8] |  | 20.8 |
| Sezer 2022 | 105 | 1.9 | [0.2; 6.7] |  | 13.8 |
|  |  |  |  |  |  |
| Fixed Effects model | 1281 | 4.5 | [3.5; 5.8] |  | 100 |
| **Random effects model** | **1281** | **4.2** | **[2.6; 6.2]** |  | **100** |
| Heterogeneity: Q=10.0; p=0.041  I^2^= 59.9%, [0.0; 85.0] | | | |  |  |

| **Knees – 12 months, affecting work and normal activity** | | | | | |
| --- | --- | --- | --- | --- | --- |
| **Author** | **Size**  (n) | **ER**  (%) | **95% CI**  [range] | **Event rate and 95% CI** | **Weight** (%) |
| Felemban 2021 | 377 | 11.1 | [8.2; 14.8] | 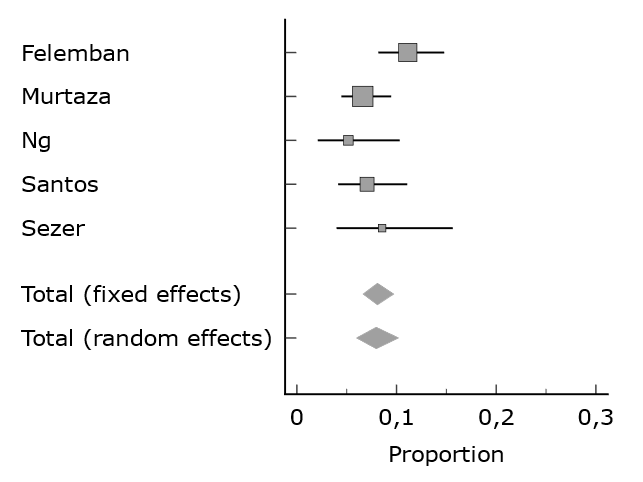 | 29.4 |
| Murtaza 2021 | 422 | 6.6 | [4.5; 9.5] |  | 32.9 |
| Ng 2016 | 136 | 5.1 | [2.1; 10.3] |  | 10.7 |
| Santos 2019 | 241 | 7.1 | [4.2; 11.1] |  | 18.8 |
| Sezer 2022 | 105 | 8.6 | [4.0; 15.7] |  | 8.2 |
|  |  |  |  |  |  |
| **Fixed Effects model** | **1281** | **8.1** | **[6.7; 9.7]** |  | **100** |
| Random effects model | 1281 | 8.0 | [6.0; 10.2] |  | 100 |
| Heterogeneity: Q=7.3; p=0.120  I^2^= 45.4%, [0.0; 80.0] | | | |  |  |

| **Ankles/Feet – 12 months, affecting work and normal activity** | | | | | |
| --- | --- | --- | --- | --- | --- |
| Author | Size  (n) | ER  (%) | 95% CI  [range] | Event rate and 95% CI | Weight (%) |
| Felemban 2021 | 377 | 10.3 | [7.5; 13.9] | 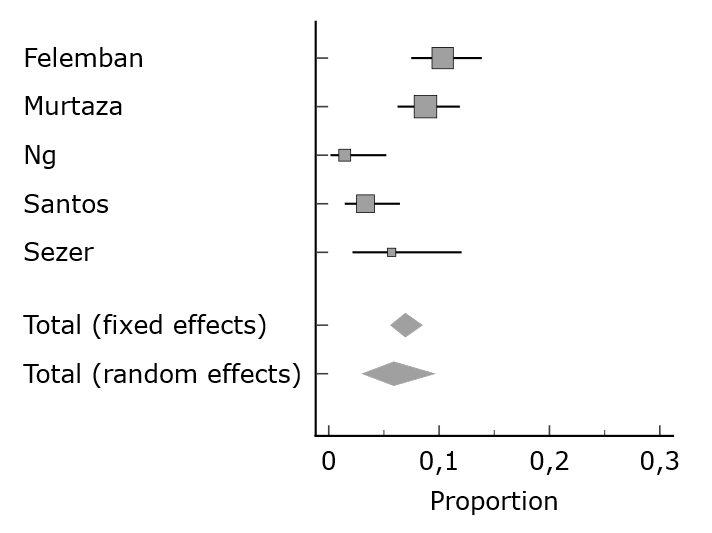 | 22.0 |
| Murtaza 2021 | 422 | 8.8 | [6.3; 11.9] |  | 22.2 |
| Ng 2016 | 136 | 1.5 | [0.2; 5.2] |  | 18.3 |
| Santos 2019 | 241 | 3.3 | [1.4; 6.4] |  | 20.6 |
| Sezer 2022 | 105 | 5.7 | [2.1; 12.0] |  | 16.9 |
|  |  |  |  |  |  |
| Fixed Effects model | 1281 | 6.9 | [5.6; 8.5] |  | 100 |
| **Random effects model** | **1281** | **5.9** | **[3.1; 9.6]** |  | **100** |
| Heterogeneity: Q=23.6; p=0.0001  I^2^= 83.1%, [61.4; 92.6] | | | |  |  |

# APPENDIX 3

Week prevalence of WRMSDs in musculoskeletal body regions, certainty assessment and level of evidence.

| **Nº of studies** | **Certainty assessment** | | | | | | |  | **Effect** | | **Level of Evidence** |
| --- | --- | --- | --- | --- | --- | --- | --- | --- | --- | --- | --- |
|  | **Design** | **Risk of bias** ^a^ | **Inconsistency** ^b^ | **Indirectness** ^c^ | **Imprecision** ^d^ | **Publication bias** | |  | **n** | **Event rate [95% CI]** |  |
|  |  |  |  |  |  | **Egger** | **Begg** |  |  |  |  |
| **7-day prevalence of WRMSDs in musculoskeletal body regions** | | | | | | | |  |  |  |  |
| **Neck** | | | | | | | |  |  |  |  |
| 10 | Observational | Not serious | 94.8% | Not serious | Serious | 0.15 | 0.06 |  | 2145 | 27.0% [19.1-35.8] | Very Low ⊕ |
| **Shoulders** | | | | | | | |  |  |  |  |
| 10 | Observational | Not serious | 95.6% | Not serious | Serious | 0.58 | 0.33 |  | 2145 | 19.6% [12.1-28.4] | Very Low ⊕ |
| **Elbows** | | | | | | | |  |  |  |  |
| 11 | Observational | Not serious | 88.0% | Not serious | Serious | 0.61 | 0.94 |  | 2247 | 3.9% [1.9-6.6] | Very Low ⊕ |
| **Hands/Wrists** | | | | | | | |  |  |  |  |
| 10 | Observational | Not serious | 94.3% | Not serious | Serious | 0.66 | 0.53 |  | 2145 | 12.0% [6.8-18.5] | Very Low ⊕ |
| **Upper-back** | | | | | | | |  |  |  |  |
| 9 | Observational | Not serious | 93.0% | Not serious | Serious | 0.24 | 0.68 |  | 1954 | 24.2% [17.2-32.0] | Very Low ⊕ |
| **Lower-back** | | | | | | | |  |  |  |  |
| 9 | Observational | Not serious | 92.7% | Not serious | Serious | 0.25 | 0.21 |  | 1954 | 27.2% [20.0-35.0] | Very Low ⊕ |
| **Hips/Thighs** | | | | | | | |  |  |  |  |
| 8 | Observational | Not serious | 84.3% | Not serious | Serious | 0.93 | 0.81 |  | 1690 | 8.9% [5.6-12.9] | Very Low ⊕ |
| **Knees** | | | | | | | |  |  |  |  |
| 9 | Observational | Not serious | 84.0% | Not serious | Serious | 0.46 | 0.53 |  | 1954 | 11.5% [8.1-15.5] | Very Low ⊕ |
| **Ankles/Feet** | | | | | | | |  |  |  |  |
| 8 | Observational | Not serious | 80.8% | Not serious | Serious | 0.99 | 0.62 |  | 1690 | 10.3% [7.1-14.1] | Very Low ⊕ |
| **12-month prevalence of WRMSDs in musculoskeletal body regions** | | | | | | | |  |  |  |  |
| **Neck** | | | | | | | |  |  |  |  |
| 13 | Observational | Not serious | 96.7% | Not serious | Not serious | 0.46 | 0.18 |  | 3101 | 51.0% [41.0-61.0] | Low ⊕⊕ |
| **Shoulders** | | | | | | | |  |  |  |  |
| 12 | Observational | Not serious | 93.1% | Not serious | Not serious | 0.87 | 0.27 |  | 2368 | 45.3% [37.6-53.1] | Low ⊕⊕ |
| **Elbows** | | | | | | | |  |  |  |  |
| 11 | Observational | Not serious | 94.6% | Not serious | Serious | 0.11 | 0.14 |  | 2191 | 7.8% [3.6-13.4] | Very Low ⊕ |
| **Hands/Wrists** | | | | | | | |  |  |  |  |
| 11 | Observational | Not serious | 91.8% | Not serious | Not serious | 0.50 | 0.70 |  | 2226 | 40.1% [33.0-47.6] | Low ⊕⊕ |
| **Upper-back** | | | | | | | |  |  |  |  |
| 13 | Observational | Not serious | 92.6% | Not serious | Not serious | 0.95 | 0.71 |  | 2484 | 38.9% [31.8-46.1] | Low ⊕⊕ |
| **Lower-back** | | | | | | | |  |  |  |  |
| 14 | Observational | Not serious | 95.3% | Not serious | Not serious | 0.68 | 0.70 |  | 3278 | 42.0% [34.0-50.2] | Low ⊕⊕ |
| **Hips/Thighs** | | | | | | | |  |  |  |  |
| 10 | Observational | Not serious | 85.7% | Not serious | Serious | 0.23 | 0.33 |  | 2049 | 13.3% [9.6-17.6] | Very Low ⊕ |
| **Knees** | | | | | | | |  |  |  |  |
| 11 | Observational | Not serious | 89.0% | Not serious | Serious | 0.73 | 0.59 |  | 2226 | 18.1% [13.4-23.3] | Very Low ⊕ |
| **Ankles/Feet** | | | | | | | |  |  |  |  |
| 10 | Observational | Not serious | 87.4% | Not serious | Serious | 0.88 | 0.93 |  | 2049 | 19.6% [14.9-24.8] | Very Low ⊕ |
| **12-month prevalence of WRMSDs in musculoskeletal body regions affecting work and normal activities** | | | | | | | |  |  |  |  |
| **Neck** | | | | | | | |  |  |  |  |
| 6 | Observational | Not serious | 73.3% | Not serious | Serious | 0.15 | 0.06 |  | 2145 | 27.0% [19.1-35.8] | Low ⊕⊕ |
| **Shoulders** | | | | | | | |  |  |  |  |
| 6 | Observational | Not serious | 80.6% | Not serious | Serious | 0.58 | 0.33 |  | 2145 | 19.6% [12.1-28.4] | Very Low ⊕ |
| **Elbows** | | | | | | | |  |  |  |  |
| 5 | Observational | Not serious | 90.4% | Not serious | Serious | 0.61 | 0.94 |  | 2247 | 3.9% [1.9-6.6] | Very Low ⊕ |
| **Hands/Wrists** | | | | | | | |  |  |  |  |
| 5 | Observational | Not serious | 88.7% | Not serious | Serious | 0.66 | 0.53 |  | 2145 | 12.0% [6.8-18.5] | Very Low ⊕ |
| **Upper-back** | | | | | | | |  |  |  |  |
| 5 | Observational | Not serious | 73.8% | Not serious | Serious | 0.24 | 0.68 |  | 1954 | 24.2% [17.2-32.0] | Very Low ⊕ |
| **Lower-back** | | | | | | | |  |  |  |  |
| 6 | Observational | Not serious | 67.6% | Not serious | Serious | 0.25 | 0.21 |  | 1954 | 27.2% [20.0-35.0] | Very Low ⊕ |
| **Hips/Thighs** | | | | | | | |  |  |  |  |
| 5 | Observational | Not serious | 59.9% | Not serious | Serious | 0.93 | 0.81 |  | 1690 | 8.9% [5.6-12.9] | Very Low ⊕ |
| **Knees** | | | | | | | |  |  |  |  |
| 5 | Observational | Not serious | 45.4% | Not serious | Serious | 0.46 | 0.53 |  | 1954 | 11.5% [8.1-15.5] | Very Low ⊕ |
| **Ankles/Feet** | | | | | | | |  |  |  |  |
| 5 | Observational | Not serious | 83.1% | Not serious | Serious | 0.99 | 0.62 |  | 1690 | 10.3% [7.1-14.1] | Very Low ⊕ |

^a^ Studies have at least a fair critical appraisal score

^b^ Serious if I^2^ > 50%

^c^ Studied population correspond to the population in study

^d^ Serious if 95% CI Range Difference > 50% of Event Rate
